# Liquid-liquid phase separation induces pathogenic tau conformations in vitro

**DOI:** 10.1038/s41467-020-16580-3

**Published:** 2020-06-04

**Authors:** Nicholas M. Kanaan, Chelsey Hamel, Tessa Grabinski, Benjamin Combs

**Affiliations:** 1 Department of Translational Neuroscience, College of Human Medicine, Michigan State University, Grand Rapids, MI 49503 USA; 2 Neuroscience Program, Michigan State University, East Lansing, MI 48825 USA; 3 Hauenstein Neuroscience Center, Mercy Health Saint Mary’s, Grand Rapids, MI 49503 USA

**Keywords:** Protein aggregation, Alzheimer's disease, Molecular neuroscience

## Abstract

Formation of membrane-less organelles via liquid-liquid phase separation is one way cells meet the biological requirement for spatiotemporal regulation of cellular components and reactions. Recently, tau, a protein known for its involvement in Alzheimer’s disease and other tauopathies, was found to undergo liquid–liquid phase separation making it one of several proteins associated with neurodegenerative diseases to do so. Here, we demonstrate that tau forms dynamic liquid droplets in vitro at physiological protein levels upon molecular crowding in buffers that resemble physiological conditions. Tau droplet formation is significantly enhanced by disease-associated modifications, including the AT8 phospho-epitope and the P301L tau mutation linked to an inherited tauopathy. Moreover, tau droplet dynamics are significantly reduced by these modified forms of tau. Extended phase separation promoted a time-dependent adoption of toxic conformations and oligomerization, but not filamentous aggregation. P301L tau protein showed the greatest oligomer formation following extended phase separation. These findings suggest that phase separation of tau may facilitate the formation of non-filamentous pathogenic tau conformations.

## Abstract (web summary)

Tau plays an important role in tauopathies and undergoes liquid-liquid phase separation (LLPS). The authors show that disease-related P301L mutant and phosphomimic (S199E/S202E/T205E) tau enhance LLPS in vitro at physiological levels, and using specific antibodies, that tau LLPS leads to pathological conformations such as N-terminal exposure and oligomeric species.

## Introduction

Compartmentalization allows for the spatiotemporal separation of biological constituents and reactions, an essential feature of cell biology. Though organelles are typically encapsulated by membranes, an alternative form of molecular organization by which specific biomolecules are localized and concentrated within discrete subcellular regions is the formation of membrane-less organelles^1^. Nucleoli, stress granules, P granules and Cajal bodies are examples of membrane-less organelles involved in multiple cellular processes including gene regulation, ribosome function and regulation of signal transduction^2^. These organelles display characteristics of liquid-like structures and are believed to form via a process called liquid–liquid phase separation (LLPS)^2^. Phase separation into liquid droplet-like structures allows rapid, reversible condensation of specific proteins and nucleic acid molecules into discrete assemblies that dynamically exchange biomolecules with the surrounding cytoplasm and nucleoplasm^3–6^. Several so-called disordered proteins and other proteins containing low complexity domains are involved in the formation of membrane-less organelles in situ^7^ and undergo LLPS into liquid droplet structures in vitro^8^. Formation of membrane-less organelles or LLPS structures has important implications for the normal biological functions and pathological processes that involve these proteins^9,10^.

Tau is a microtubule-associated protein that is traditionally described as “intrinsically disordered” and shares many of the same properties as other proteins that phase separate and are found in membrane-less organelles^11^. Tau proteins exhibit concentrated regions of charged residues, distributed aromatic (i.e. tyrosine and phenylalanine) and polar amino acids and segments with highly negative or positive overall charges at physiological pH. Moreover, tau is present in membrane-less organelles, including the nucleolus and stress granules where it may serve normal functional roles and potentially pathological roles in disease^12–14^. Given these characteristics of tau, it is not entirely surprising that multiple studies demonstrate tau phase separation in vitro at physiological and supraphysiological levels under a relatively wide range of experimental conditions^15–28^, and recent evidence suggest tau may participate in phase separation-like structures and/or stress granules in neurons and other cells^14,19,24,29^. Some of these studies suggest that the physiological role of tau phase separation may include regulation of RNA, stress granule formation and/or nucleating microtubule polymerization (reviewed in refs. ^30,31^). Moreover, data suggest tau phase separation is influenced by electrostatic interactions between different subdomains of the protein, the lysine amino acid composition of tau, post-translational modifications of tau (e.g. phosphorylation and acetylation), extension of the polypeptide chain and/or interactions with other proteins (e.g. tubulin and EFhd2) or RNAs^15–28^. These findings support a potential role for tau as a component of LLPS structures, a hypothesis that would align with the accumulating evidence that tau is more than simply a microtubule stabilizing protein.

Tau became infamous for its role in several diseases collectively known as tauopathies, including Alzheimer’s disease (AD) and numerous forms of frontotemporal dementia (e.g. frontotemporal dementia with parkinsonism linked to chromosome 17, FTDP-17)^32,33^. These diseases are characterized by abnormal and pathogenic changes to tau conformation, oligomerization and aggregation of the protein, as well as increased tau phosphorylation^34^. Several of these modifications confer toxic properties onto tau. Conformational display of the phosphatase-activating domain (PAD) in the extreme amino terminus^35^, formation of soluble oligomeric species^36^ and the presence of the AT8 phosphoepitope^37,38^ confer toxicity on tau via a mechanism that impairs axonal transport^35^ and synaptic functions^39,40^. Mutation of proline 301 to a leucine in tau (P301L) is particularly toxic and causes inherited FTDP-17 in humans^41^. The processes that drive the transition of tau from a normal monomer to pathological forms remain unknown, as do the factors that modulate tau LLPS and whether tau LLPS generates pathogenic tau species.

Here, we show that full-length, wild-type, human tau undergoes LLPS at physiological levels (i.e. 1–2 µM) that is dependent upon the presence of both the N terminus and microtubule-binding domains (MTBRs). We also found that disease-related P301L and AT8 modifications significantly enhance LLPS in vitro. Importantly, we demonstrate that prolonged LLPS leads to reduced droplet dynamics and a time-dependent increase in specific pathogenic conformations and oligomeric tau species that are linked to mechanisms of tau toxicity without the appearance of bona fide tau filaments. Collectively, these data highlight a potential role for tau LLPS in the formation of neurotoxic tau species and provide insights into the effects that disease-related modifications have on tau phase separation in vitro.

## Results

### Strategy for studying tau liquid–liquid phase separation

A common method for studying LLPS is to either conjugate fluorophores to the protein of interest or to create fluorescent protein fusion constructs. Tau exhibits two well-characterized in vitro behaviors, microtubule binding and aggregation into filaments. Before using fluorescently labeled proteins in LLPS studies, we determined whether fluorescent labels affect microtubule binding and in vitro tau aggregation behavior (Supplementary Methods). We assessed the microtubule-binding ability of recombinant, full-length human tau (tau; 441 amino acids, the hT40 or 2N4R isoform), tau-GFP fusion protein (tau-GFP), Tau labeled with fluorescein isothiocyanate (FITC-tau) or tau labeled with Alexa 568 dye (A568-tau). Binding to microtubules with tau and tau-GFP was robust as evidenced by nearly complete pull-down of tau with microtubules in pelleting assays (Supplementary Fig. 1a, b). In contrast, FITC-tau and A568-tau showed reduced microtubule binding (Supplementary Fig. 1a, b). These data indicate that tau-GFP fusion protein and unlabeled tau bind microtubules similarly, whereas conjugation of fluorescent molecules impedes microtubule binding.

We determined whether fluorescent labels affect in vitro aggregation behavior with arachidonic acid induction. Normally, tau forms a mixture of filamentous aggregates and small globular oligomeric aggregates^42^. As expected, unlabeled tau produced the typical mixture of filaments and globular oligomers, which was nearly identical to the aggregates formed by tau-GFP proteins (Supplementary Fig. 1c). In contrast, FITC-tau and A568-tau produced large clusters of clumped proteins that did not resemble the standard filamentous aggregates formed by arachidonic acid induction (Supplementary Fig. 1c). Both FITC-tau and A568-tau can phase separate into droplet-like structures (Supplementary Fig. 1d, e); however, their impaired microtubule binding and abnormal in vitro aggregation properties raise concern of whether these proteins are appropriate for analysis of tau behavior in vitro. Thus, tau-GFP fusion proteins were used in subsequent LLPS imaging experiments because they exhibit normal microtubule binding and aggregation in vitro, criteria often not employed in prior studies using fluorescently labeled tau proteins to study phase separation.

### Characteristics of tau liquid droplets

Clusters of positively and negatively charged amino acids, as well as distributed tyrosine and phenylalanine residues, are important for facilitating LLPS of proteins through multivalent electrostatic interactions^7^. The tau protein is a highly flexible disordered protein with highly charged domains and amino acid composition favorable for facilitating the interactions within and between proteins that allow LLPS. Recent reports indicate that full-length human tau and truncated tau constructs undergo liquid phase separation at a range of supraphysiological levels^15–20^ and at near physiological levels^21–28^. The physiological level of tau in neurons is ~2 µM^43^, therefore, we performed our experiments with full-length human tau proteins at 2 µM tau unless otherwise noted.

**Fig. 1:**
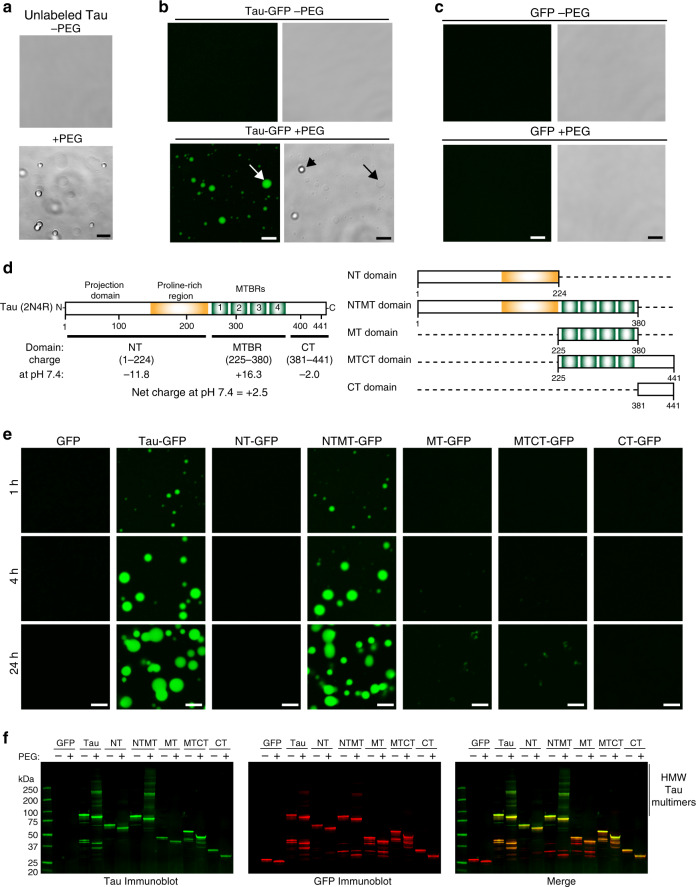
Tau undergoes liquid–liquid phase separation at physiological concentrations. **a** Unlabeled tau (2 μM) does not underdo LLPS without molecular crowding (−PEG), but under crowding conditions (i.e. +PEG at 10%) full-length human tau forms liquid droplets. Scale bar is 5 µm. **b** Similarly, tau-GFP undergoes phase separation at physiological levels (2 µM tau) to form spherical liquid droplet structures under molecular crowding conditions (+PEG), not in the absence of crowding (−PEG). Tau-GFP liquid droplets initially are formed suspended in solution (black arrowhead), and once settled on the glass slide they wet the surface (white and black arrows). Scale bars are 5 µm. **c** Recombinant GFP (2 µM) does not form liquid droplets whether PEG is present or not demonstrating that GFP is unlikely to drive tau-GFP LLPS. Scale bars are 5 µm. All experiments performed in LLPS buffer ±10% PEG. **d** Diagrams of full-length human tau protein illustrating the charges of various domains of the protein at pH 7.4 (overall protein charge is +2.7, note the NT (aa 1–224) = −11.8, and the MT (aa 225–380) = +16.3) and the CT (aa 381–441) = −2.0. The tau domain proteins used in this study also are depicted. **e** To study which tau domains undergo phase separation, GFP fusion proteins of specific tau domains were used, including the N terminus alone (NT, aa 1–224), the NT through the MTBRs (NTMT, aa 1–380), the MTBRs alone (MT, aa 225–380), the MTBRs through the C terminus (MTCT, aa 225–441) and the CT alone (aa 381–441). The only tau domain construct that exhibited phase separation into liquid droplets at 2 μM was the NTMT protein even after 24 h suggesting the MT and CT are not sufficient for phase separation (representative images of three independent experiments). Notably, the MT alone and MTCT proteins showed a time-dependent (primarily after 24 h) formation of small irregularly shaped structures that did not resemble liquid droplets. As expected GFP alone did not phase separate or form any structures even after 24 h incubation indicating GFP is unlikely to be responsible for the behavior of the tau proteins. **f** Immunoblotting of GFP and tau domain proteins incubated without (−; i.e. monomeric proteins) or with 10% PEG (+; i.e. liquid droplets) for 4 h shows a clear presence of heat-, reducing- and SDS-stable high molecular weight tau multimers only in full-length tau (tau) and NTMT tau proteins incubated with PEG. Note the other domains did not undergo liquid droplet formation and did not effectively form such multimers confirming these tau species are specifically associated with phase separation. Source data provided in the Source Data file.

We first established whether unlabeled full-length tau formed liquid droplets in a standard HEPES buffer (LLPS buffer: 10 mM HEPES, 150 mM NaCl, 0.1 mM EDTA, 2 mM DTT, pH 7.4) with and without 10% polyethylene glycol (PEG) as a crowding agent (referred to as LLPS buffer from here). At 2 µM, unlabeled tau formed spherical liquid droplets in the presence of 10% PEG, but not when PEG was absent (Fig. 1a). Full-length tau-GFP (2 µM) also formed spherical, floating liquid droplets only under molecular crowding conditions and wetted the glass surface after settling to the bottom of the dish over time (Fig. 1b). The reproducibility of this effect between protein preparations was confirmed by making three independent tau-GFP preparations and all exhibited the same behavior (Supplementary Fig. 1f). Finally, we tested GFP alone (2 µM) for liquid droplet formation to determine whether GFP was likely responsible for this behavior with tau-GFP proteins. GFP alone (2 μM) did not form liquid droplets in the presence or absence of PEG for up to 4 (Fig. 1c) or 24 h (Fig. 1e), and did not show signs of phase separation up to 8 µM with PEG (Supplementary Fig. 1g). At pH 7.4, full-length tau has an overall net charge of +2.5, but there is a notable dipolar charge arrangement with the N-terminal domain (aa 1–224) having a −11.8 charge and the C-terminal half (containing the MTBRs and C terminus, aa 225–441) having a +14.3 charge (Fig. 1d). These data establish that at physiological levels tau undergoes phase separation to form liquid droplets independent of GFP.

To further establish which regions of tau are required for droplet formation, we divided tau into specific domains, including the N terminus alone (NT-GFP, aa 1–224), the NT with the MTBRs (NTMT-GFP, aa 1–380), the MTBRs alone (MT-GFP, aa 225–380), the C terminus with the MTBRs (MTCT-GFP, aa 225–441) and the C terminus alone (CT-GFP, aa 381–441). At pH 7.4, the overall net charge of the NT is −11.8, the NTMT is +4.5, the MT is +16.3, the MTCT is +14.3 and the CT is −2.0 (Fig. 1d). We found that the liquid droplet formation was observed only when the NT and MTs were present together (i.e. full-length and NTMT tau), indicating that in isolation the NT, MT, MTCT or CT do not undergo phase separation under the conditions used (i.e. 2 μM, +10% PEG for up to 24 h; Fig. 1e). Interestingly, proteins composed of the MT alone or the MTCT appeared to form small irregularly shaped structures over time (being most notable after 24 h), which did not resemble the liquid droplets formed with full-length tau and NTMT (Fig. 1e). The samples were further analyzed using immunoblotting to assess whether heat-, reducing- and SDS-stable high molecular weight tau multimers, which are characteristic of pathological tau multimerization, were formed after 4 h of phase separation into liquid droplets (Fig. 1f). The only proteins that effectively formed stable tau multimers (i.e. bands above the respective monomer bands) were the full-length tau protein and the NTMT tau protein (Fig. 1f), which are the two that underwent liquid droplet formation and showed no sign of filamentous aggregate formation (Fig. 1e).

**Fig. 2:**
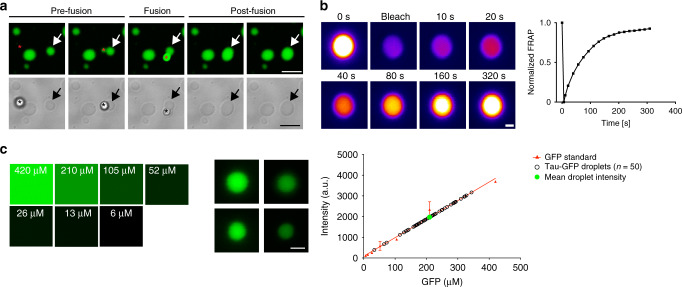
Liquid droplet-like properties of phase-separated tau structures. **a** Tau-GFP (2 µM) phase-separated structures undergo fusion events, which are characteristic of liquid droplets. Scale bars are 5 µm. **b** An illustrative example of tau-GFP fluorescence recovery after photobleaching (FRAP), which was used to confirm the dynamic nature of tau liquid droplets (see Fig. 5 for additional FRAP data). Scale bar is 1 µm. **c** Tau-GFP (2 µM) concentration within liquid droplets was estimated at an average of 210.7 μM (SD = ±71.82, Min = 33.9 μM, Max = 343.9 μM) after 2 h incubation (*n* = 50 droplets). Images of recombinant GFP protein at a range of 6–420 μM were used for a standard curve of GFP fluorescence signal (linear regression, *r*^2^ = 0.98). Scale bar is 5 µm. The red data points and line are the GFP standard, open black circles are the individual droplets measured and the green circle indicates the mean tau-GFP droplet intensity. Data are from a representative experiment that was repeated three times with similar results. All experiments in LLPS buffer +10% PEG. Source data for **b** and **c** provided in the Source Data file.

Phase-separated tau structures exhibit additional liquid droplet characteristics including droplet fusion, dynamic exchange of proteins with the surrounding environment and increased molecule concentration within droplets. We found that tau-GFP (2 µM) liquid droplets readily fused (Fig. 2a). tau-GFP droplets showed rapid recovery after bleaching as assessed by fluorescence recovery after photobleaching (FRAP), indicating dynamic exchange of proteins between the droplet and the surrounding soluble proteins (a representative tau-GFP droplet is shown in Fig. 2b). We estimated the concentration of tau in liquid droplets at 210.7 μM (range: 33.9–343.9 µM) by using recombinant GFP to make a standard curve for fluorescence intensity and measuring the intensity of individual tau-GFP droplets (2 μM; Fig. 2c). Thus, the estimated total concentration of tau in droplets is ~100 times the level of the soluble proteins in the bulk phase solution. This demonstrates that phase-separated tau structures formed under crowding conditions exhibit key characteristics of liquid-like droplets in vitro.

**Fig. 3:**
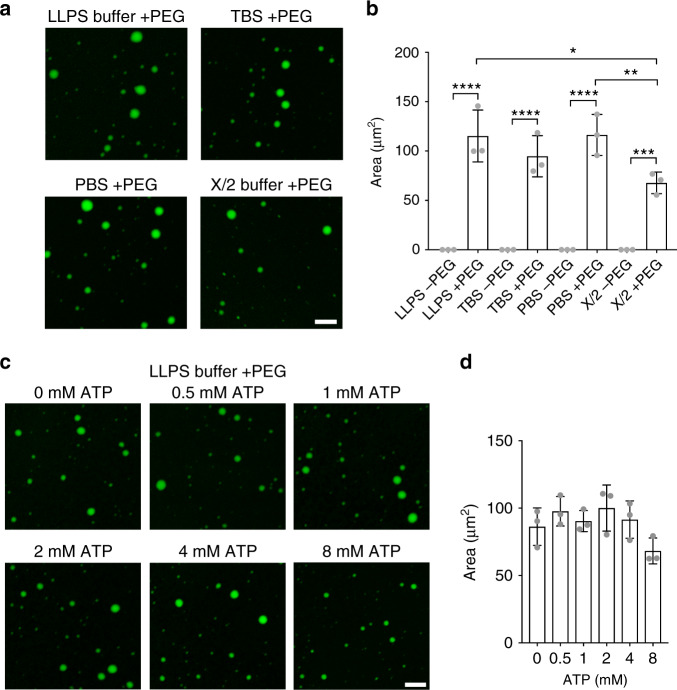
Tau phase separates into liquid droplets in multiple buffers. **a** Tau-GFP (2 µM) forms liquid droplet in the LLPS buffer, tris buffered saline, phosphate-buffered saline and X/2 buffer under crowding conditions, not in the absence of crowding. Scale bar is 5 µm. **b** The extent of droplet formation was significantly higher in LLPS and PBS buffers compared to X/2 buffer (graph represents mean ± SD; *n* = 3 independent experiments; ****p < 0.001, ***p = 0.0005, **p = 0.0098, *p = 0.0109, two-way ANOVA with Holm–Sidak post-hoc test). **c** Tau-GFP (2 µM) forms liquid droplets under crowding conditions (+PEG) across the range of physiological levels of ATP (0–8 mM) in the LLPS buffer. Scale bar is 5 µm. **d** ATP (0–8 mM) does not significantly impact the amount of tau-GFP droplet formation (graph represents mean ± SD; *n* = 3 independent experiments; *p* = 0.095, one-way ANOVA). Source data for **b** and **d** provided in the Source Data file.

Next, we established whether phase separation into liquid droplets occurred in different buffers used for in vitro studies. We chose LLPS buffer (detailed above), tris buffered saline (TBS; tris 50 mM, NaCl 150 mM, pH 7.4), phosphate-buffered saline (PBS; KH_2_PO_4_ 1.5 mM, Na_2_HPO_4_ 8 mM, CaCl_2_ 1 mM, MgCl_2_ 0.5 mM, KCl 2.7 mM, NaCl 140 mM, pH 7.4) and X/2 buffer (HEPES 10 mM, potassium aspartate 175 mM, taurine 65 mM, betaine 35 mM, glycine 25 mM, MgCl_2_ 6.46 mM, EGTA 5 mM (stock adjusted to pH 7.2 with KOH), CaCl_2_ 1.5 mM, glucose 0.5 mM, ATP 1 mM (stock adjusted to pH 7.2 with KOH)). TBS and PBS are commonly used reagents, whereas the X/2 buffer was chosen because it was developed to resemble the physiological composition of axoplasm^44^. Tau-GFP remained as a dispersed monomeric solution without crowding, but tau liquid droplets formed under molecular crowding (i.e. 10% PEG) in all of the buffers tested (Fig. 3a). The LLPS buffer and PBS buffers showed significantly more droplet formation compared to the X/2 buffer (Fig. 3b). Recently, Patel and colleagues^45^ showed that ATP can act as a hydrotrope that disrupts phase separation of some proteins. Given that it is the most abundant nucleotide in the cytoplasm^46^, we tested whether ATP alters tau droplet formation in LLPS buffer. Over a range of ATP concentrations (i.e. 0–8 mM) there was little to no effect on tau phase separation (Fig. 3c, d). Thus, tau condenses into liquid droplets in several buffers, including those that mimic characteristics of the intracellular milieu (e.g. ionic and ATP composition, pH, reducing strength, biomolecular crowding, etc.).

Collectively, these data demonstrate that tau readily undergoes LLPS to form highly concentrated tau droplets (~100 times the bulk phase) at physiological tau concentrations (i.e. 2 µM) in numerous buffers, including those that approximate physiological intracellular composition.

### Disease-related tau enhances droplets and lowers dynamics

**Fig. 4:**
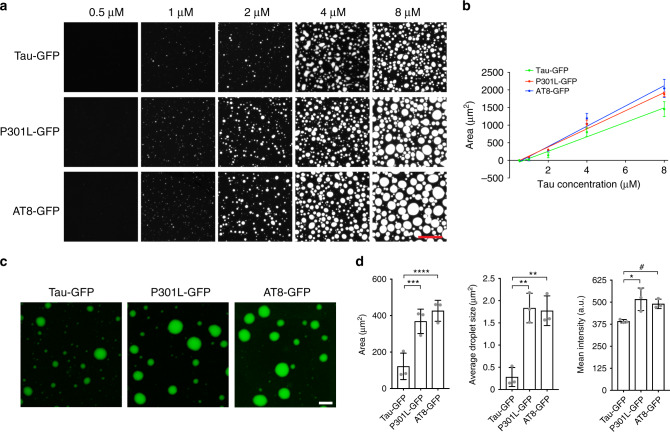
Disease-related tau modifications significantly enhance liquid droplet formation. **a** Images of tau-GFP, P301L-GFP, AT8-GFP liquid droplets with a range of protein concentrations (0.5–8 μM) after 2 h incubation under crowding conditions. Scale bar is 40 µm. **b** Linear regression analysis for estimating the critical droplet concentration (i.e. *x*-intercept) for each tau construct (graph represents mean ± SD; *n* = 3 independent experiments). The CDC for P301L-GFP (0.53 μM; *r*^2^ = 0.99) and AT8-GFP (0.63 μM; *r*^2^ = 0.98) were slightly lower than the CDC of tau-GFP (0.74 μM; *r*^2^ = 0.98), but the differences did not reach statistical significance (*p* = 0.32, one-way ANOVA). **c** Images of tau-GFP, P301L-GFP and AT8-GFP (all at 2 µM) liquid droplets under crowding conditions after 1 h incubation. Scale bar is 2 μm. **d** The extent of droplet formation (total area), individual droplet size and droplet fluorescence intensity were significantly higher in P301L-GFP and AT8-GFP droplets compared to tau-GFP droplets (graph represents mean ± SD; *n* = 3 independent experiments; one-way ANOVA with Holm–Sidak post-hoc test, ****p = 0.0039, ***p = 0.0075, **p = 0.0021, *p = 0.0272, ^#^*p* = 0.0474). All experiments in LLPS buffer +10% PEG. Source data for **b** and **d** provided in the Source Data file.

Tauopathies are marked by pathological changes in the tau protein, whether it be mutations associated with inherited tauopathies (e.g. P301L mutant tau in FTDP-17)^33^ or post-translational modifications such as increased phosphorylation (e.g. the AT8 phospho-tau site)^37,38^. Similar to tau-GFP, both P301L-GFP and AT8-GFP (i.e. tau with pseudophosphorylation mutations S199E/S202E/T205E at sites recognized by the phospho-specific AT8 antibody) form liquid droplets only under crowding conditions (Fig. 4). We confirmed that each tau-GFP fusion protein preparation was comparable between the different tau constructs (Supplementary Fig. 2). The critical droplet concentration (CDC) of tau-GFP, P301L-GFP and AT8-GFP was determined by measuring the extent of droplet formation with a range of tau (i.e. 0.5–8 μM). The CDC was estimated at 0.74 µM for tau-GFP, 0.53 µM for P301L-GFP and 0.63 µM for AT8-GFP (Fig. 4a, b). Image analysis was used to quantify the differences between the liquid droplets formed by each tau construct at 2 µM. The total amount of liquid droplets formed (i.e. total area), individual droplet size and mean fluorescence intensity of droplets formed by P301L-GFP and AT8-GFP were significantly greater than tau-GFP droplets at 2 µM (Fig. 4c, d). These data indicate that P301L and AT8 significantly enhance tau phase separation and the lower limit of droplet formation for all three tau proteins is <1 µM or just below the physiological levels of tau in neurons^43^.

**Fig. 5:**
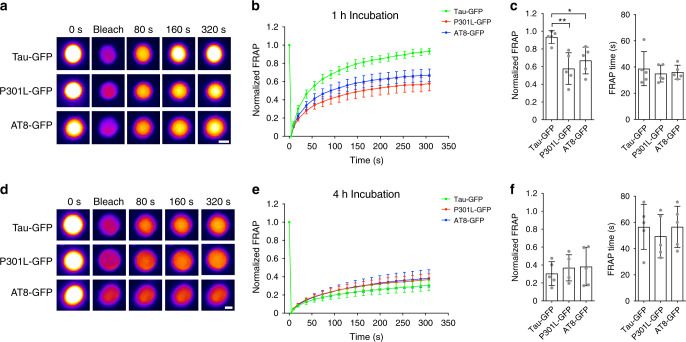
Disease-related tau modifications form liquid droplet with reduced dynamic properties. **a** Representative droplets of tau-GFP, P301L-GFP and AT8-GFP (4 µM each, 2 µM tau-GFP and 2 µM tau unlabeled) analyzed using FRAP after 1 h incubation in crowding conditions. **b** FRAP curves for each tau protein after 1 h incubation indicate that each protein shows dynamic exchange of proteins with the surrounding bulk phase (graph represents mean ± SEM). **c** Comparison of the final extent of recovery after photobleaching (i.e. 320 s) demonstrates that tau-GFP liquid droplets show significantly more recovery than P301L-GFP and AT8-GFP droplets after 1 h incubation (one-way ANOVA with Holm–Sidak post-hoc test, **p = 0.0054, *p = 0.0237, graph represents mean ± SD). The FRAP time was not statistically different between tau constructs (*p* = 0.24, one-way ANOVA). **d** Representative droplets of tau-GFP, P301L-GFP and AT8-GFP (4 µM each, 2 µM tau-GFP and 2 µM tau unlabeled) analyzed using FRAP after 4 h incubation in crowding conditions. **e** FRAP curves for each tau protein analyzed after 4 h incubation demonstrate marked reductions in the dynamic properties of droplets from all three tau proteins (graph represents mean ± SEM). **f** Comparison of the final extent of recovery after photobleaching (i.e. 320 s) for all tau liquid droplets show similar low levels of recovery after 4 h incubation (*p* = 0.30, one-way ANOVA, graph represents mean ± SD). The FRAP time was not statistically different between tau constructs (*p* = 0.31, one-way ANOVA). The 1 and 4 h FRAP experiments were repeated five independent times. Source data for **b**, **c**, **e** and **f** provided in the Source Data file.

Next, we determined whether disease-related modifications of tau impacted the dynamic properties of phase-separated tau liquid droplets. First, the dynamics of tau liquid droplets were measured with FRAP after 1 h incubation. Tau-GFP displayed higher dynamic properties compared to P301L-GFP and AT8-GFP (Fig. 5a–c). The immobile phase of tau-GFP (6.6%) is approximately six and five times lower than P301L-GFP (42.2%) and AT8-GFP (33.0%), respectively. However, all three forms of tau display similar FRAP kinetics (i.e. FRAP time; tau-GFP=38.7 s; P301L-GFP=35.0 s; AT8-GFP=36.1 s). After 4 h of incubation, the dynamics of all tau proteins tested are substantially reduced (Fig. 5d–f). The immobile phase was similar for tau-GFP (69.5%), P301L-GFP (63.1%) and AT8-GFP (61.8%), as were the FRAP kinetics (tau-GFP=56.6 s; P301L-GFP=49.5 s; AT8-GFP=56.7 s). These findings demonstrate that disease modifications of tau (i.e. mutant P301L tau and phospho-AT8 tau) facilitate the formation of less dynamic liquid droplets when compared to wild-type tau protein.

### Tau phase separation leads to pathological tau conformations and oligomerization

**Fig. 6:**
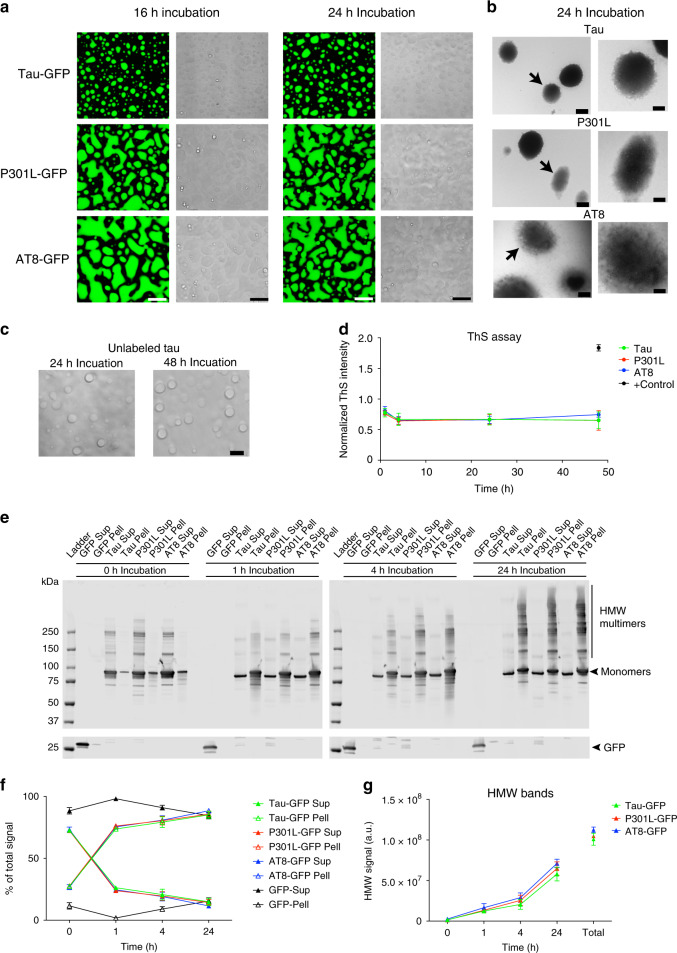
Prolonged incubation of tau liquid droplets produces multimeric tau species but does not lead to fibril formation. **a** Images of phase-separated structures formed by tau-GFP, P301L-GFP and AT8-GFP (each at 4 µM) after 16 and 24 h incubation at room temperature. Scale bar is 5 µm. **b** Transmission electron micrographs of phase-separated structures from unlabeled tau, P301L and AT8 proteins (4 µM each) after 24 h incubation at room temperature. Tau filaments were not readily apparent in the structures formed under these conditions (arrows indicate the droplet shown in the higher magnification images). Scale bars are 800 nm (left images) and 400 nm (right images). This experiment was repeated three independent times. **c** Incubation of unlabeled tau for 24 and 48 h produces droplets, not fibrillar structures, further confirming the lack of fibril formation via LLPS. **d** Thioflavin S (ThS), a fibrillar cross-β-sheet binding dye that labels tau filaments, showed low signal and no increase over time (1–48 h, data normalized to LLPS buffer with PEG blank) with prolonged phase separation confirming the lack of cross-β-sheet structures in tau proteins (e.g. filaments) (graph represents mean ± SD). Note that the positive control samples, arachidonic acid-induced tau aggregates (black circle), showed a robust increase in ThS intensity. **e** Supernatant (Sup) and pelleted (Pell) fractions from droplet spin down experiments were run in western blots to assess the time-dependent (0–24 h of phase separation) formation of heat-, reducing- and SDS-stable tau multimers. Notably, some high molecular weight tau species (HMW, i.e. tau multimers) were apparent shortly after addition of PEG (0 h time point). **f** Quantification of total tau in the Sup or Pell fractions shows a clear time-dependent shift in pelletable tau starting 1 h after phase separation is initiated and continuing through 24 h (graph represents mean ± SD). **g** Quantification of the HMW tau multimers shows a time-dependent increase in stable multimers with prolonged phase separation with all tau proteins (graph represents mean ± SD). Source data for **d**–**g** provided in the Source Data file.

Previous reports using RNA/DNA binding proteins, such as fused in sarcoma (FUS) and heterogeneous nuclear ribonucleoprotein A1 (hnRNPA1), found that prolonged incubation in liquid droplet-inducing conditions facilitates the formation of fibrils^8,47–50^. We examined whether prolonged incubation of tau in crowding conditions leads to fibril formation using 4 µM tau (2 µM tau-GFP and 2 µM unlabeled tau) to provide more tau protein in the longer experiments. Incubation of tau for 16 and 24 h produced substantial amounts of phase-separated structures that were amorphous for all three tau constructs (Fig. 6a), but there was no evidence of fibril-like structures using confocal microscopy imaging. We further confirmed the lack of fibril formation with unlabeled tau, P301L and AT8 after 24 h in crowding conditions using electron microscopy (Fig. 6b). Next, we assessed whether filaments form after extended phase separation (24 and 48 h) using unlabeled tau protein and again did not observe fibril-like structures using microscopy (Fig. 6c). Moreover, we performed thioflavin S (ThS) fluorescence assays, a standard fibrillar cross-β-sheet dye assay of tau fibrilization, and did not observe evidence of enhanced ThS fluorescence from 1 to 48 h after phase separation (Fig. 6d). Finally, we performed droplet pelleting assays to isolate phase-separated droplets and determine whether there was evidence that LLPS leads to formation of stable multimers, which are heat-, reducing- and SDS-resistant multimers characteristic of pathological tau species, in the absence of filament formation (Fig. 6e, f). Indeed, extended phase separation showed a time-dependent increase in the amount of tau stable multimers formed by tau-GFP, P301L-GFP and AT8-GFP tau proteins in the pellet fraction that are resistant to heat, biochemical reduction and SDS detergent. Thus, prolonged incubation in crowding conditions clearly leads to formation of stable tau multimers, but does not lead to obvious tau fibril formation under the conditions tested.

**Fig. 7:**
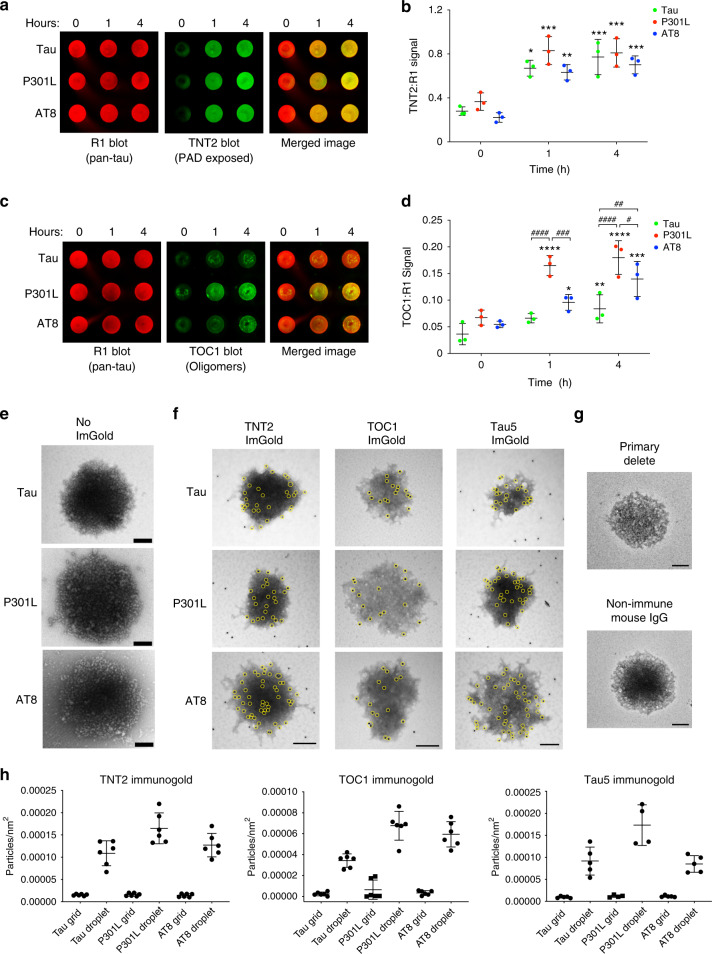
Phase separation leads to formation of pathological tau conformations and oligomeric tau. **a** Unlabeled tau, P301L and AT8 tau proteins incubated in LLPS buffer for 0, 1 or 4 h (each at 4 µM) were dot blotted and probed for total tau (R1 antibody) and PAD-exposed tau (TNT2 antibody). **b** Quantitation of dot blots show significant increases in PAD exposure upon incubation of tau, P301L and AT8 proteins with PEG for 1 and 4 h compared to 0 h (*n* = 3 independent experiments; two-way ANOVA with Sidak post-hoc test ***p < 0.0001, **p = 0.0001, *p = 0.0002 compared to respective 0 h time point; graphs are mean ± SD). **c** Representative dot blot images of unlabeled tau, P301L and AT8 tau proteins incubated for 0, 1 or 4 h in LLPS buffer and probed for total tau (R1 antibody) and oligomeric tau (TOC1 antibody). **d** Quantitation of dot blots show significant time-dependent increases in oligomeric tau species upon LLPS of tau, P301L and AT8 with significantly more TOC1 reactivity in the P301L and AT8 samples compared to tau (*n* = 3 independent experiments; two-way ANOVA with Sidak post-hoc test, ****p < 0.0001, ***p = 0.0003, **p = 0.0402, compared to respective 0 h time point, *p = 0.0420 compared to respective 0 and 4 h time point, ^####^*p* < 0.0001, ^###^*p* = 0.0017, ^##^*p* = 0.0095, ^#^*p* = 0.0328; graphs are mean ± SD). **e** Transmission electron micrographs of negatively stained liquid droplets composed of unlabeled tau, P301L tau and AT8 tau (4 µM, 4 h incubation). Scale bars are 200 nm. **f** Immunogold labeling of tau, P301L and AT8 liquid droplets (4 µM, 4 h incubation) confirm the presence of PAD-exposed tau (TNT2 antibody) and oligomeric tau (TOC1 antibody) within droplets. A pan-tau antibody (Tau5) confirmed the structures are composed of tau. Yellow circles are around gold particles for easier visualization and scale bars are 200 nm. **g** Primary antibody delete and non-immune mouse IgG (substituted as the primary antibody) controls confirm the specificity of the immunogold labeling in **f**. Scale bars are 200 nm. **h** Quantitation of gold particle density demonstrates a clear increase in labeling (~10-fold) with each antibody within droplet when compared to the grid, confirming the specificity for labeling within droplets (graph represents mean ± SD). Source data for **a**–**d**, **h** provided in the Source Data file.

Tau filaments are the major constituent of mature tau pathologies (e.g. neurofibrillary tangles), while pre-fibril conformational changes in tau, such as exposure of the PAD in the extreme amino terminus and tau oligomerization, are linked more directly to mechanisms of tau toxicity^35,51^. As LLPS clearly induced multimer formation in a time-dependent fashion (Fig. 6), we next determined whether prolonged LLPS of tau led to the formation of PAD-exposed tau and/or tau oligomers, using two monoclonal antibodies. TNT2 is an antibody directed against the PAD sequence (epitope is aa 7–12) and in non-denaturing assays (e.g. dot blots) this reagent is a useful marker of whether PAD is conformationally displayed in tau^52^. TOC1 is an antibody that was made against tau dimers (epitope is aa 209–224) and selectively binds tau oligomers (not monomers or filaments) in non-denaturing assays^53,54^. Neither TNT2 or TOC1 react with soluble recombinant tau monomers or soluble tau derived from nondemented control brain samples, suggesting they represent disease-associated pathogenic species of tau^35,52,53,55–57^. First, we confirmed that each unlabeled tau protein preparation was comparable between the different tau constructs (Supplementary Fig. 2). Then, we determined whether liquid droplet formation of unlabeled tau, P301L, and AT8 would affect TNT2 and TOC1 reactivity after 0, 1 and 4 h incubation by using dot blots. Incubation with PEG for 1 and 4 h led to a significant increase in TNT2 signal with tau, P301L and AT8 proteins when compared to the 0 h time point, but TNT2 signal after 1 and 4 h was similar among all three proteins (Fig. 7a, b). TOC1 signal showed a time-dependent increase with all three tau proteins, an effect that was slower in tau (i.e. only reaching significance after 4 h) than P301L and AT8 (Fig. 7d). P301L tau displayed significantly more TOC1 compared to tau and AT8 at both the 1 and 4 h time points, whereas AT8 showed significantly more reactivity compared to tau at the 4 h time point (Fig. 7d). We confirmed that the significant increase in TNT2 and TOC1 epitopes was not simply an effect of incubating tau, P301L and AT8 samples for 4 h at room temperature by comparing samples incubated with and without PEG for 4 h (Supplementary Fig. 3). There was a significant increase in the amount of PAD-exposed tau (Supplementary Fig. 3a, b) and oligomeric tau (Supplementary Fig. 3c, d) in +PEG samples when compared to −PEG tau samples confirming the requirement of crowding (i.e. PEG). These data suggest LLPS conditions cause PAD exposure in tau proteins and a time-dependent increase in tau oligomer formation, an effect that was exacerbated by both AT8 and P301L modifications.

Tau droplets formed after 4 h incubation in crowding conditions were imaged using negative staining and electron microscopy. The droplets from all three proteins were spherical in shape and appeared to contain densely packed globular structures (Fig. 7e). We next used immunogold labeling of total tau, PAD-exposed tau and oligomeric tau to confirm whether the tau proteins specifically within liquid droplets were positive for these two pathological conformations (Fig. 7f). The total tau antibody, Tau5, revealed that spherical structures formed under crowding conditions are composed of tau (Fig. 7f). Probing with the TNT2 and TOC1 antibodies (Fig. 7f) confirmed that tau within the liquid droplets is in conformations with PAD exposed and as multimeric oligomers, respectively. There was some degree of TNT2 labeling of tau outside of the liquid droplet structures indicating that some monomeric or small order multimers of tau proteins also display PAD in +PEG conditions (Fig. 7f). Both primary delete and non-immune mouse IgG (in place of primary antibody) controls did not produce gold particle labeling of the droplets (Fig. 7g), confirming the specificity of the immunogold staining. To further confirm the specificity of labeling and enrichment of TNT+ and TOC1+ conformations in droplets the density of gold particles within droplets and on the grid was quantified (Fig. 7h). All densities of gold particles on the grid were very low, whereas each tau maker (TNT2, TOC1 and Tau5) showed clear enrichment (~10-fold) in tau, P301L and AT8 tau droplets (Fig. 7h). It is noteworthy that occasionally “spoke-like” structures appeared to emanate from droplets only in the immunogold labeling experiments, not the unlabeled grids used above, which is likely due to the use of unfixed samples and extensive processing of the grids required for immunolabeling (as outlined in the Methods). Together, these data demonstrate that tau undergoing LLPS can adopt pathological conformations that expose PAD and form oligomers, both of which are associated with tau toxicity independent of filament formation^35,37^.

## Discussion

Compartmentalization through both membrane-bound and membrane-less organelles are essential features of the structure-function relationship in cells. Thus, better understanding the factors involved in forming membrane-less organelles through LLPS is critical to advancing our understanding of cell biology, as well as the pathobiology of some diseases. We demonstrate that tau forms phase-separated liquid droplets in vitro, a finding consistent with recent studies using either full-length tau or various truncated constructs and several crowding or inducing agents (e.g. PEG, dextrans, ficoll and RNAs (poly(A), poly(U) and tRNA))^15–28^. In our hands, phase-separated tau structures also showed key characteristics of liquid-like droplets including spherical shape, dynamic exchange with the surrounding bulk phase, droplet fusion and surface wetting. Moreover, we found that tau phase separation required the N-terminal domain and the MTBRs, as isolated N-terminal, MTBR or C-terminal domains did not form liquid droplets nor did a protein containing the MTBR and C-terminal domains. Our findings compliment those from most studies using recombinant full-length tau proteins^15,16,19,21,23,25–28^, as well as those from Boyko et al.^25^ that found tau liquid droplet formation was dependent upon electrostatic interactions between the N terminus and MTBRs of tau. Notably, Ukmar-Godec and colleagues also found that tau lacking the N terminus does not form droplets with dextran crowding, but does with poly(U) RNA to a lesser extent than full-length tau^19^. Our data do not align with a study showing that recombinant full-length tau (from *E. coli*) does not undergo LLPS with PEG crowding^24^, and other studies showing that tau proteins lacking the N terminus or proteins consisting only of the N terminus form liquid droplets^16–18,20,22,24,27^. The reasons underlying these discordant results are not entirely clear, but likely are due to differences in the amount of tau used (e.g. physiological vs supraphysiological) and/or specific experimental conditions (e.g. protein source, buffer conditions, or temperature).

We show that unmodified, full-length human tau undergoes LLPS at physiological levels in conditions that approximate physiological composition and crowded environment of the cytoplasm. At 2 µM tau, liquid droplet formation required molecular crowding (i.e. 10% PEG) and was quite robust as droplets formed in the presence of ATP (up to 8 mM) and in several buffers. It is noteworthy that tau undergoes LLPS with crowding in X/2 buffer, which is designed to mimic axoplasmic composition. Importantly, the estimated CDC for liquid droplet formation in vitro was just under 1 µM, which is similar to other reports^23,24,26,27^ and is less than half of the physiological levels of tau in neurons (i.e. ~2 μM)^43^. These findings suggest that normal neuronal concentrations of full-length human tau are sufficient for LLPS. This compliments previous studies that demonstrate full-length tau and truncated domains containing the MTBRs undergo LLPS at similar physiological levels and at a large range of supraphysiological levels (e.g. >4–500 µM)^15–28^. We also establish that liquid droplets can generate a robust local increase in tau concentration, with our estimates at ~100 times the surrounding bulk phase solution. The previous findings, and those reported here, clearly indicate that phase separation of tau is a physiochemical property of tau under conditions that approximate the physiological environment of cells. However, the potential effects of disease-related modifications on LLPS of full-length tau were not well established.

The intracellular mechanisms driving the transition of tau from a functional soluble protein to a pathological form continue to elude the field. An emerging picture among the LLPS literature is that phase separation is potentially tied to disease-related forms of proteins involved in multiple neurodegenerative diseases and may provide a mechanism for the formation of pathological conformations and/or aggregates of these proteins. Recent studies suggest phosphorylation^22,24^ and acetylation^23,26,28^ alter the propensity of tau to undergo phase separation. We examined the effects of the P301L mutation and phospho-AT8 tau, two prominent disease-related forms of tau, on LLPS behavior. Both forms of tau significantly enhanced tau phase separation, resulting in droplets that displayed slower dynamics compared to wild-type tau. Moreover, with increasing time the dynamic exchange of all three tau proteins in and out of the droplets was significantly reduced indicating that prolonged phase separation leads to more static tau structures. These findings resemble the enhanced phase separation and aggregation of P301L tau^24^ and reduction in dynamics of mutant FUS and mature hnRNPA1 droplets^8,49^. Pelleting experiments show that phase separation leads to a progressive accumulation of tau in pelletable structures (i.e. liquid droplets) as well as heat-, reducing- and SDS-stable multimers. We also used biochemical assays to demonstrate that prolonged tau LLPS in vitro facilitates the time-dependent appearance of PAD exposure and oligomeric tau conformations. The adoption of PAD-exposed conformations during prolonged phase separation was relatively similar between wild-type and disease-related forms of tau. Interestingly, the adoption of the TOC1 conformation showed a clear increase with AT8 tau that was further increased with P301L tau over time, suggesting these modifications specifically facilitate formation of oligomeric structures with prolonged phase separation. Transmission electron microscopy (TEM) showed that the ultrastructure of liquid droplets appears to be densely packed globular structures, and importantly, immunolabeling electron microscopy confirmed that PAD exposed and oligomeric tau exists within liquid droplets. The implications of the above findings are significant because these forms of tau are directly linked to mechanisms of tau toxicity (reviewed in^58^).

Exposure of the PAD signaling motif in disease-related forms of tau triggers a signaling pathway involving protein phosphatase 1 (PP1) and glycogen synthase kinase 3 (GSK3) that causes cargoes to dissociate from the kinesin-1 motor protein complex, effectively disrupting axonal transport^59^. PAD-dependent tau toxicity also was observed in synaptic transmission^40^ and a recent study showed that Aβ-induced toxicity in primary neurons requires tau-mediated activation of GSK3β^60^. Importantly, pathological modifications, such as oligomerization and AT8 phosphorylation (mimicked via pseudophosphorylation), led to enhanced PAD exposure, directly linking these modifications to this mechanism of tau toxicity^35,55^. Indeed, tau oligomers appear early in human disease^54^, are diffusible and cause neuronal dysfunction and toxicity through disruption of synaptic function (i.e. long-term potentiation and long-term depression)^61,62^, disruption of mitochondrial function^63^ and axonal transport impairment^37^. Likewise, AT8 phosphorylation is an early modification that appears as tau begins depositing in human AD brains and directly interferes with axonal transport without the requirement of aggregation/oligomerization^35,64^. In addition, there is a connection between tau oligomerization, cell toxicity and stress granule formation, a membrane-less organelle thought to be created through phase separation-like processes. Tau facilitates the formation of stress granules where it interacts with RNA binding proteins such as T-cell intercellular antigen 1 (TIA1), which promotes tau oligomerization and neurotoxicity in cultured cells and in vivo^14,29,65–67^. Interestingly, acetylation of tau may represent a modification that mitigates tau phase separation in vitro and incorporation into stress granules in cells^19,26^. Thus, published data and evidence reported here suggest tau phase separation (potentially into stress granules) represent one mechanism by which pathogenic conformations are adopted in situ and might contribute to neurotoxicity.

The molecular forces driving the observed differences in LLPS behavior and conformational changes between wild-type tau and the two disease-associated forms tested here are not entirely clear. However, the P301L mutation makes tau more susceptible to pathogenic conformational changes that may underlie impaired tau functionality^68^ and facilitates aggregation by elongating β-strand structures^69^ but does not alter the charge of the protein. AT8 phosphorylation increases the negative charge in the amino terminal half of tau (addition of −3.0 charge), and alters its conformation by disrupting normal interactions between the N terminus and the C terminus, known as the “paperclip” conformation^70,71^. Thus, it is likely that effects on the structure of tau could facilitate stronger intermolecular interactions that underlie the enhanced liquid droplet formation of P301L and AT8 tau. Indeed, several pathological conformations, such as the Alz50/MC1 conformations, aberrant PAD exposure and oligomeric conformations, are related to pathogenic changes in tau that are thought to toxicity.

Tau oligomers and PAD exposure were evident in tau liquid droplets, but we did not find clear evidence of traditional tau filament formation (either by confocal microscopy, electron microscopy or ThS assays) with the tau proteins tested. This finding held true despite continued accumulation of tau within droplets, formation of stable multimers, and extended incubations of 48 h. These data agree with recent reports that LLPS of the full-length or MTBR tau constructs did not form fibrillar cross-β-sheet structures, as indicated by low to no ThT or ThS signal, despite robust phase separation^16,17,22,27^. It is noteworthy that many studies on tau phase separation have not noted the formation of fibrillar structures under wide ranging experimental conditions, although specific experiments to this point were not provided^15,18–21,23,25,26,28^. The appearance of PAD-exposed and oligomeric tau species, but not stable fibrillar cross-β-sheet structures, is consistent with previous observations that the early deposition of TNT2 and TOC1-reactive tau pathology in human brain tissue does not colocalize with thiazine red (a similar cross-β-sheet dye)^52,54^. We estimated the concentration of tau protein in liquid droplets at ~200 µM (i.e. 100 times the bulk phase levels), suggesting that simply increasing tau concentration is not sufficient to overcome the energy barriers preventing bona fide filament formation, at least in vitro. Tau protein is normally maintained in a phosphorylated state with ~1–3 mol phosphate per mol of tau, and disease-associated forms of tau have an average of ~6–8 mol phosphate per mol tau^72–74^. Tau filament formation may require additional tau modifications or other factors, such as arachidonic acid or heparin, to form filamentous aggregates^22,42^. Indeed, the most robust evidence of tau LLPS leading to potential fibril-like aggregates with cross-β-sheet structures was seen using heavily phosphorylated (~12 mol phosphate/mol tau^75^) tau proteins purified from Sf9 insect cells, whereas MARK2 phosphorylated bacterial-derived tau and a recombinant 17× phospho-mimetic tau protein did not show apparent fibril structure formation in the same study^24^. Alternatively, LLPS-induced multimers may not represent “on-pathway” structures for directly seeding tau filaments, but instead is a mechanism by which soluble protein structures facilitate adoption of toxic conformations independent of filament formation. Future studies are necessary to further test this hypothesis.

**Fig. 8:**
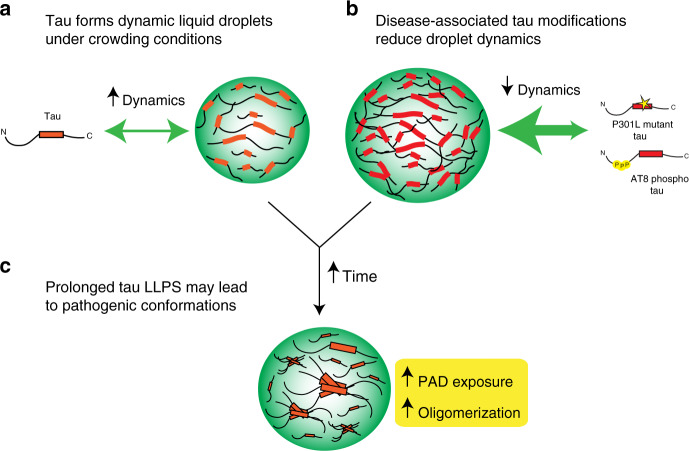
Proposed hypothetical model of tau liquid–liquid phase separation in normal and pathological conditions. **a** Under normal physiological conditions, phase separation could lead to the formation of tau liquid droplet-like structures that show high dynamics. **b** Forms of tau associated with disease, such as phosphorylated tau (e.g. at the AT8 site) or mutant tau that causes inherited tauopathies, facilitate liquid droplet formation. **c** Pathological conditions that facilitate tau phase separation and/or prolong phase separation may lead to the adoption of pathological tau conformations (i.e. PAD exposure and oligomerization) that have been linked to cell toxicity.

Disease-related mechanisms are often an aberration of a normal function and, while LLPS is only a recently discovered physiochemical property of tau protein, it may have important implications for normal and pathological tau mechanisms. Although replicating physiological processes in vitro is inherently difficult, such studies may effectively provide starting points to further investigate potential biological events that occur in situ. On the basis of our in vitro findings, we propose a working hypothetical model of the relationship between LLPS of tau and disease-related tau modifications. Under normal conditions, tau could readily transition between soluble monomers and phase-separated liquid droplets, which likely assemble and disassemble rapidly and exhibit high dynamics with the surrounding cytoplasm or nucleoplasm (Fig. 8a). Tau liquid droplets could facilitate specific functions related to the discrete cellular compartments (e.g. nucleoli, axons, soma, etc.) in which they form. Known disease-related forms of tau, including mutant P301L tau that causes inherited tauopathies and phospho-AT8 tau seen in several tauopathies, enhance formation of liquid droplets that are less dynamic (Fig. 8b). Neurons in patients with tau mutations such as P301L may face sustained enhancement of tau LLPS because of the mutation, whereas cell stress and injury can increase pathological AT8 phospho-tau^76,77^. Sustained or dysregulated phase separation is potentially problematic because this leads to the adoption of multimeric tau conformations, such as PAD exposure and oligomerization (Fig. 8c), both of which are directly linked to neurotoxic mechanisms and do not appear in normal soluble tau^35,52,53,55–57^.

## Methods

### Recombinant tau and GFP proteins

Seven recombinant full-length proteins were made for this work, including full-length wild-type human tau (tau, hT40 or 2N4R tau), P301L mutant tau, pseudophosphorylated (S199E, S202E and T205E) AT8 tau, tau fused with C-terminal GFP (tau-GFP), P301L fused with GFP (P301L-GFP), AT8 fused with GFP (AT8-GFP) and GFP protein alone. Resources used in this study are summarized in Supplementary Table 1. As a validation step, we performed multiple independent productions of proteins (e.g. 3 preps of tau-GFP) to ensure phase-separation behavior was not an artifact of a single preparation (Supplementary Fig. 1f). In addition, five tau domain constructs consisting of N terminus alone (NT-GFP, aa 1–224), the NT with the MTBRs (NTMT-GFP; aa 1–380), the MTBR alone (MT-GFP; aa 225–380), the MTBR with the C terminus (MTCT-GFP; aa 225–441) and the CT alone (CT-GFP; aa 381–441) were created with C-terminal GFP fusions as well (Fig. 1d). All of the recombinant proteins had a C-terminal 6× poly-histidine tag to facilitate purification and were made using a combination of standard mutagenesis, PCR and/or cut-and-paste cloning methods (Supplementary Table 2). All plasmids were sequence verified using standard Sanger sequencing (Genewiz) prior to use in protein production (plasmid maps were made using DNAStar Sequence Builder software (v15)) and sequence alignment/verification was done using DNAStar Sequence Manager software (v15). Each protein was grown in T7 express *E. coli* cells and proteins were purified following published detailed protocols^78^. Briefly, cells were induced to express proteins with 1 mM IPTG, followed by collection of cells and lysis of the cell pellet (lysis buffer: 500 mM NaCl, 10 mM Tris, 5 mM imidazole, pH 8.0 with freshly added 1 mM PMSF and 10 μg/ml each of pepstatin, leupeptin, bestatin and aprotinin). Proteins were extracted through a three-step chromatography process with a GE Akta Pure system (running Unicorn software v6.4). First, proteins were captured on a Talon metal affinity resin column (Talon wash buffer: lysis buffer from above; Talon elution buffer: 500 mM NaCl, 10 mM Tris, 100 mM imidazole, pH 8.0 with freshly added 0.8 mM PMSF), the resulting proteins were further cleaned using size exclusion chromatography with an S500 column (S500 buffer: 100 mM Tris, pH 7.4), and finally the samples were further polished over an HiTrap Q anion exchange column (elution buffer: 100 mM Tris, pH 7.4 and a 0–250 mM NaCl linear gradient). After purification, 1 mM DTT was added to the protein stocks, which were aliquoted and stored at −80 °C until used for experiments. We confirmed that each recombinant tau protein preparation (unlabeled and GFP fusion tau proteins) were comparable in quantity and purity using standard SDS-PAGE and Coomassie staining methods (Supplementary Fig. 2a, b). Coomassie gel images were acquired using a BioRad Gel Doc EZ Imager equipped with Image Lab software (v5.2.1). Recombinant tau (without GFP tag) was conjugated to FITC or AlexaFluor 568 (Supplementary Methods and Supplementary Fig. 1a–e).

### Tau liquid droplet reactions

Tau LLPS was examined in the absence and presence of 10% polyethelene glycol (PEG, Sigma, 81269, PEG 3000 monodisperse solution, used for ≤6–8 months) as a crowding agent. In our hands, tau did not form liquid droplets in the absence of crowding conditions even at 8 µM (four times physiological levels of tau in neurons) with 4 h incubation. Thus, all liquid droplet samples were generated under crowding conditions (i.e. 10% PEG). Several buffers were tested, including standard HEPES buffer (LLPS buffer: 10 mM HEPES, 150 mM NaCl, 0.1 mM EDTA, 2 mM DTT, pH 7.4), tris buffered saline (TBS; tris 50 mM, NaCl 150 mM, pH 7.4), phosphate-buffered saline with magnesium and chloride (PBS; KH_2_PO_4_ 1.5 mM, Na_2_HPO_4_ 8 mM, CaCl_2_ 1 mM, MgCl_2_ 0.5 mM, KCl 2.7 mM, NaCl 140 mM, pH 7.4) and X/2 buffer (HEPES 10 mM, potassium aspartate 175 mM, taurine 65 mM, betaine 35 mM, glycine 25 mM, MgCl_2_ 6.46 mM, EGTA 5 mM (stock adjusted to pH 7.2 with KOH), CaCl_2_ 1.5 mM, glucose 0.5 mM, ATP 1 mM (stock adjusted to pH 7.2 with KOH)). The majority of studies utilized the LLPS buffer, unless otherwise noted. To test the effects of ATP on tau LLPS, ATP was added to the LLPS buffer with 10% PEG at a range of concentrations (0–8 mM). Unlabeled tau (2 µM) was used to confirm that tau without fluorescent labels undergoes phase separation in LLPS buffer with and without PEG. All liquid droplet samples used for microscopy were performed with tau-GFP fusion proteins as indicated. Recombinant GFP (2–8 μM) was used as a control to confirm that GFP did not drive LLPS of tau-GFP constructs. For all LLPS reactions, 10% PEG was added last by simultaneously swirling the sample and gently pipetting up and down to thoroughly mix the sample.

### Confocal microscopy image and FRAP analysis

The samples imaged with confocal microscopy were added to glass bottom 8-well chamber slides to image at ×60 magnification (oil lens, 1.4 numerical aperture) on a Nikon A1+ confocal system with Nikon Elements Imaging Software (v5). Images were taken at the indicated times and tau proteins were at the indicated concentrations. Droplets were imaged with a 488 nm laser and 450/50 nm detection filters and the transmitted light channel.

Image analysis was used to measure the extent of liquid droplet formation, droplet size and droplet fluorescence intensity using FIJI Image J software (v2.0). The fluorescence images within a set of experiments were acquired using identical microscope settings (i.e. scan speed, resolution, magnification, optical zoom, gain, offset and laser intensity) to ensure consistency across samples and experimental runs. Thresholding was set to a range of 360–4095 gray levels, which effectively eliminated the background signal from the analysis (Supplementary Fig. 4). The pixels within these threshold limits were evaluated for total area (μm^2^, equivalent to the amount of droplets), average object size (μm^2^, equivalent to droplet size) and average fluorescence intensity of each object (arbitrary units, equivalent to the amount of tau-GFP proteins within droplets). The GFP fluorescence standard was performed by imaging solutions of GFP protein at 420, 210, 105, 52, 26, 13 and 6 µM in tris buffered saline (TBS; 50 mM tris, 150 mM NaCl, pH 7.4). Using the same acquisition settings (i.e. scan speed, resolution, magnification, optical zoom, gain, offset and laser intensity), images were taken of tau-GFP (2 μM) droplets to estimate the intra-droplet concentration of tau. Several droplets (~50 per experiment) were analyzed by drawing a circular region of interest around the droplet to obtain the average pixel intensity for each droplet. Linear regression analysis of the intensity of GFP standard images (*r*^2^ = 0.99) was used to interpolate the estimated concentration of tau proteins within droplets. Although highly linear and capable of estimating droplet tau concentrations and providing a measure relative differences between the tau protein droplets under the same conditions, this method is not quantitative and thus will not provide absolute concentrations of tau within droplets.

FRAP was used to measure the dynamic exchange of proteins between the droplets and the surrounding solution of soluble proteins after a short (i.e. 1 h) and long (i.e. 4 h) incubation time. To provide additional tau proteins into the reaction the FRAP studies included half tau (unlabeled) and half tau-GFP protein (e.g. a 4 µM tau-GFP reaction used for FRAP analysis had 2 µM tau-GFP+2 µM tau). At ×60 magnification and ×6 optical zooming, droplets were outlined with a circular “bleaching” overlay object (~8 per field of view), an unbleached droplet was circled with a “reference” overlay object and a circular “background” overlay object was placed in an area without droplets. The FRAP parameters were as follows: a single pre-bleaching image (T0), bleaching at laser power set at 40 and 1 frame/2 s rate, an immediate post-bleach image and several images over a total of 4 min (figures display T80, T160 and T320 second images). The FRAP curves were calculated by normalizing fluorescence signal to the background and reference signals and FRAP time (i.e. time to half maximal recovery signal) was determined using these functions in the Nikon Elements Software (v5). The immobile fraction was determined as the difference between the initial droplet intensity and the intensity of the droplet at the final time point (i.e. T320 seconds). All imaging was done with the same acquisition settings (i.e. scan speed, resolution, magnification, optical zoom, gain, offset and laser intensity). The FRAP experiments were repeated five independent times with the intensities from all droplets analyzed in each experiment (i.e. 10–40 droplets per experiment) averaged together to obtain one of the five data points.

### TEM and immunogold labeling

Samples were prepared on formvar coated copper mesh grids for standard negative stain TEM imaging as described previously^78^. TEM does not require GFP fusion proteins, thus, we used unlabeled tau for these experiments and to increase the amount of droplets on each TEM grid these experiments were performed using 4 µM tau and the samples were unfixed in these studies. Standard negative stained TEM grids were prepared using unfixed samples and minimal processing to observe the ultrastructural morphology of tau droplets. Briefly, grids were rinsed in a drop of dH_2_0, the samples were prepared by putting a droplet (20 µl) of sample on the inside of the cap of a 1.5 ml microcentrifuge tube (filled with 100 µl H_2_O in the bottom of the tube to humidify the tube) and then the grids were placed on the droplets. The tube was closed leaving the grid at the bottom of the sample droplet to allow the phase-separated structures to settle onto the grid. The grids were incubated at room temperature for either 4 or 24 h and processed for negative staining. After incubation with the samples, the grids were rinsed once in water, rinsed once in 2% uranyl acetate and then incubated on a drop of uranyl acetate for 2–3 min before drying and imaging on a JEOL 1400+ TEM system equipped with a TEM Center Software (v1.5) and AMT Image Capture Engine Software (v602).

Immunogold labeling TEM grids of unfixed samples were prepared to localize specific tau conformations within droplets, but more extensive processing is required. For immunogold labeling and TEM imaging, tau liquid droplet samples (4 μM) formed for 4 h were adsorbed onto formvar coated nickel mesh grids as above. Grids were then rinsed in 1× tris buffered saline (TBS; 150 mM NaCl, 50 mM Tris, pH 7.4) twice for 1 min each, then blocked with 20 µl drops of 2 mg/ml bovine serum albumin (BSA) for 10 min. The grids were then incubated for 30 min on 20 µl drops of primary antibodies diluted in 2 mg/ml BSA solution. The R1 antibody^79^ (rabbit polyclonal pan-tau antibody; AB_2832929) was used at 1:50 to label tau, the TNT2 antibody^52^ (mouse IgG1 monoclonal, AB_2736931) labels PAD-exposed tau and was used at 1:50, and the TOC1 antibody^54^ (mouse IgM monoclonal, AB_2832939) labels oligomeric tau and was used at 1:25. Two controls were used for TEM immunogold staining. A sample was processed without the presence of primary antibody (i.e. primary delete) and a sample was processed with non-immune mouse IgG (Jackson ImmunoResearch, 015-000-003, AB_2337188) in place of the anti-tau primary antibody (all other steps were identical). Neither control conditions produce immunogold labeling of tau liquid droplets confirming the specificity of the signal with Tau5, TNT2 and TOC1 immunogold labeling (Fig. 7g). Following primary antibody incubations, the grids were rinsed once in 5× TBS for 2 min, 5× TBS for 1 min and then 1× TBS for 1 min each prior to incubation in 18 nm-labeled goat anti-mouse IgG (H + L) secondary antibody (Jackson ImmunoResearch, 115-215-146, AB_2338738) diluted 1:50 in 2 mg/ml BSA solution. Then the grids were rinsed following the same steps used after the primary antibody followed by one rinse in dH_2_O, one rinse in 2% uranyl acetate and then incubated for 3–4 min in uranyl acetate. The grids were dried and imaged on a JEOL 1400+ TEM system. Images were taken at ×40,000 magnification, and areas with “positively” stained droplets were used for imaging due to the ability to more clearly delineate gold particles (Fig. 7f), which are difficult to image in droplets that are well stained with uranyl acetate (Fig. 7e). Images were analyzed by manually counting all gold particles within liquid droplets or on the grid and expressed as a density (particles/nm^2^)

### Droplet pelleting assay

To determine whether tau accumulated in droplets overtime and whether heat-, reducing- and SDS-stable multimeric species of tau were formed following phase separation, we performed sedimentation assays by pelleting. GFP, tau-GFP, P301L-GFP and AT8-GFP samples were spun at 2500×*g* for 5 min after 0 (immediately after PEG addition), 1, 4 and 24 h post-incubation with 10% PEG as above. The supernatants were collected and pellets resuspended in TBS. Samples were prepared for SDS-PAGE and western blotting as below. These experiments were repeated four independent times.

### Thioflavin S fluorescence assay

The fibrillar cross-β-sheet dye, thioflavin S (ThS), was used to assess the extent to which tau filaments formed during phase separation using methods similar to those published^55,57^. Notably, ThS does not readily bind monomeric β-sheet structures or all amyloid fibrillar structures, but is commonly used to detect tau fibrillar cross-β-sheet structures. Stocks of 0.04% ThS were made in LLPS buffer with 10% PEG (as above) fresh for each assay. Tau LLPS reactions were made as above using 2 μM unlabeled tau, P301L and AT8 in LLPS buffer containing 10% PEG. Fluorescence signal was measured using a Beckman Coulter DTX 880 Multimode Detector (SoftMax Pro6 Software, v6.3) with a 485/20 nm excitation filter and a 535/25 nm emission filter. Fluorescence intensity was read for each well prior to addition of ThS to obtain background fluorescence readings. At 1, 4, 24 and 48 h after the addition of PEG, the ThS (final concentration 0.02%) was added and fluorescence measured. A LLPS buffer with 10% PEG blank sample was used at each time point to obtain the background ThS fluorescence intensity with PEG present. Tau containing samples were normalized to the blank signal (i.e. LLPS buffer with PEG). We included a pre-aggregated wild-type tau positive control generated with arachidonic acid as described in Supplementary Methods. These experiments were repeated 4 independent times.

### Dot blotting and western blotting

Dot blot analysis was used as a non-denaturing assay to measure the extent of PAD exposure and oligomer formation following LLPS. These biochemical assays were done using 4 µM tau (unlabeled) to increase detection of tau conformations. Tau, P301L and AT8 samples were made in LLPS buffer and incubated for 0, 1 and 4 h. Separate samples were made for each time point within an experiment to allow dilution of the entire reaction prior to dot blotting and the samples were blotted fresh (i.e. without being frozen). After the indicated incubation, the samples were diluted to 17 ng/µl (100 µl/spot) in LLPS buffer, and then immediately pulled through the dot blot manifold onto a nitrocellulose membrane. The blot was immediately blocked in 2% non-fat dry milk (NFDM) for 30 min, incubated overnight at 4 °C in a primary antibody solution containing either R1^79^ (1:10,000; AB_2832929) and TNT2^52^ (1:10,000; AB_2736931) or R1 and TOC1^54^ (1:5,000; AB_2832939). Then the membranes were rinsed, incubated in a secondary antibody solution containing either goat anti-rabbit 800 (Licor, 926-32211, AB_621843) and goat anti-mouse IgG1-specific 680 (Licor, 926-68050, AB_2783642) secondary antibodies each diluted 1:20,000 (for R1 and TNT2 blots) or goat anti-rabbit 800 (Licor, 926-32211, AB_621843) and goat anti-mouse IgM AlexaFluor 680 (Jackson ImmunoResearch, 115-625-075, AB_2338934) secondary antibodies each diluted 1:20,000 (for R1 and TOC1 blots) for 1 h at room temperature. The blots were then rinsed and imaged on the Licor system and analyzed using Licor Image Studio Software (v5). The signal of TNT2 or TOC1 was ratioed to the R1 signal for each spot (to normalize to total tau levels) and these data were used for statistical comparisons. The dot blot experiments were repeated three independent times.

Standard SDS-PAGE western blotting was performed similar to previous descriptions^55,57^. Briefly, samples (400 ng/lane) were prepared in Laemmli buffer and heated to 60 °C for 5 min prior to running on a 4–20% TGX acrylamide Criterion gel (BioRad, 5671095) and transferred to 0.22 μm nitrocellulose. Membranes were blocked for 1 h in 2% NFDM in TBS followed by overnight incubation with Tau5 primary antibody (a pan-tau antibody, epitope residues 210–230, 1:100,000; AB_2721194)^80,81^ and rabbit anti-GFP (Abcam, ab290, 1:30,000; AB_303395) diluted in 2% NFDM. For tau domain blots, the membranes were probed with rabbit anti-GFP (Abcam, ab290, 1:30,000; AB_303395), and a mouse IgG1 cocktail containing Tau12 (an N-terminal antibody, aa 8/9 and 13–21, 1:500,000; AB_2721192)^52,82^, MTBR3 antibody (Kanaan lab, 1:10,000; AB_2832994; see Supplementary Methods and Supplementary Fig. 5) and Tau7 (a C-terminal antibody, aa 430–441, 1:500,000; AB_2721195)^83^. The following day, blots were rinsed in TBS containing 0.1% tween20 and then incubated in goat anti-mouse IgG1 680 (Licor, 926-68050, AB_2783642) and goat anti-rabbit 800 (Licor, 926-32211, AB_621843) secondary antibodies each diluted 1:20,000 in 2% NFDM. Blots were imaged using the Licor Odyssey system and the Licor Image Studio Software (v5.2.5) was used to quantify the signal intensity for the bands.

### Statistical analysis

Standard linear regressions were used to interpolate the intensity of tau-GFP droplets from the recombinant GFP intensity standard and to determine the critical droplet concentration for tau-GFP, P301L-GFP and AT8-GFP. Results from the LLPS buffer with ATP experiments, the comparison between tau constructs (at 2 μM) experiments, and the FRAP measurement experiments were analyzed using a one-way ANOVA and pairwise comparisons were made using the Holm–Sidak post-hoc test. Buffer comparison data (factor 1 = buffer; factor 2 = ±PEG) and dot blotting data (factor 1 = time; factor 2 = tau protein) were compared using a two-way ANOVA and pairwise comparisons between groups were made using the Sidak post-hoc test. The significance cutoff was set at *p* ≤ 0.05 for all comparisons. Linear regressions were also used to estimate the critical droplet concentration (i.e. CDC) of tau, and a non-linear regression (sigmoidal dose–response with variable slope) was used to analyze MTBR3 antibody titer data. GFP intensity standard curve data were assessed for outlier replicates (>3 times the mean and >2 standard deviations of other replicates), and accordingly a single replicate was removed from the GFP concentration standard curve imaging data (Fig. 2c, see Source data file). No other replicate or experimental data points were excluded from analysis in this work. All data were analyzed and graphed using GraphPad Prism (v8.4.1, GraphPad Software Inc.).

### Reporting summary

Further information on research design is available in the Nature Research Reporting Summary linked to this article.

## Supplementary information

Supplementary Information

Reporting Summary

## Data Availability

The source data underlying Figs. 1f, 2b, c, 3b, d, 4b, d, 5b, c, e, f, 6d–g, 7a–d, h and Supplementary Figs 1a, b, 2a, b, 3a–d, 5a, b are provided in the Source Data file. Other data are available from the corresponding author upon reasonable request.

## Source data

Source Data
