# Supplementary material for: Liquid-liquid phase separation induces pathogenic tau conformations in vitro
Source: Nat Commun. 2020 Jun 4;11:2809. doi: 10.1038/s41467-020-16580-3 (PMC7272632; doi:10.1038/s41467-020-16580-3)
Supplement: Supplementary file 1 — Supplementary Information [file 41467_2020_16580_MOESM1_ESM.pdf]

Supplementary Information

Liquid-Liquid Phase Separation Induces Pathogenic Tau Conformations In Vitro

Nicholas M. Kanaan et al.

Supplementary Methods

Fluorescent Labeling of Tau

Purified recombinant Tau was labeled with FITC and Alexa568 to establish the best approach for studying tau liquid droplet formation. First, tau protein (1 mg/ml) was buffer exchanged into 1x phosphate buffered saline using Micro Bio-Spin 6 columns (BioRad, 7326200) per the manufacturer's instructions. The proteins were then labeled with FITC (FITC-Tau, Thermo F6434) or Alexa568 (A568-Tau, Thermo, A20184) following the protocols provided by the manufacturers. Briefly, FITC labeled tau protein was created by adding 20 μl of 1M sodium bicarbonate buffer (from kit) to 200 μl of tau protein, then 10 μl of the reactive FITC dye (10 mg/ml stock in DMSO) was added while gently stirring the sample to mix, followed by incubation at room temperature for 1 hr (protected from light). After labeling, the protein sample was cleaned of free dye and buffer exchanged into PBS (pH 7.2, with 2mM sodium azide) using the spin columns provided following the kit instructions. AlexaFluor568 labeled tau protein was generated by adding 10 μl of 1M sodium bicarbonate buffer (from kit) to 100 μl of tau protein, then 100 μl of this solution was added to reactive AlexaFluor 568 dye and gently inverted to mix, followed by incubation at room temperature for 1 hr (protected from light) with gentle inversion to mix every 10 minutes. After labeling, the protein sample was cleaned of free dye and buffer exchanged into PBS (pH 7.2, with 2mM sodium azide) using the spin columns provided following the kit instructions. The labeling efficiency was calculated at 0.6 – 0.7 M dye per M of tau protein.

Arachidonic Acid-Induced Tau Aggregation *In Vitro*

In vitro aggregation of Tau, Tau-GFP, FITC-Tau and A568-Tau (all at 2 μM) was induced using arachidonic acid (75 μM) in tau polymerization buffer (100mM NaCl, 10mM HEPES (pH 7.6), 5mM DTT and 0.1mM EDTA) as described in detail previously[1]. Aggregated tau samples were imaged using standard electron microscopy imaging as detailed below[1].

Microtubule Binding Assay and Western Blotting

The effects of labeling tau with GFP or conjugating tau to fluorescent molecules (i.e. FITC and Alexa 568) on microtubule binding *in vitro* was assessed using the Microtubule Binding Protein Spin-Down Assay according to the manufacturer's instructions (Cytoskeleton, BK029). Briefly, microtubules (5 µM tubulin dimers) were generated at 35˚ C for 20 minutes and then 20 µM taxol was added to stabilize the microtubules. The samples tested included microtubules only (no tau) and either unlabeled Tau, Tau-GFP, FITC-Tau or A568-Tau (all at 0.5 µM final concentration) without and with microtubules (in a total reaction volume of 50 µl). These samples were incubated for 30 minutes at room temperature while binding took place and then spun over a sucrose cushion (100 µl, supplemented with taxol) at 100,000 x g at room temperature for 40 minutes. The supernatant was collected for each sample (50 µl, 10 µl of 6x Laemmli buffer was added), the cushion was discarded and the pellet was collected by adding 50 µl 1x Laemmli buffer.

Standard SDS-PAGE and Western blotting analyses as described previously[2] were used to evaluate results of the microtubule binding assay. Briefly, samples were heated at 95° C for 5 minutes before being separated on 26-well BioRad precast Criterion 4-20% TGX gels for ~30 minutes at 250 V. The supernatant and pellet fractions were run for each sample. The transfer was 50 minutes at 400 mA and then the membranes were blocked in 2% non-fat dry milk for 30 minutes. The blots were then incubated in a mixture of R1 antibody[3] (a rabbit polyclonal pan-tau antibody; AB_2832929) diluted 1:10,000 and 5H1 antibody[4] (a β-tubulin antibody, IgM mouse monoclonal; AB_2832941) diluted 1:15,000 in 2% non-fat dry milk overnight at 4° C. The following day, the blots were rinsed in TBS with 0.1% tween-20 and then incubated for 1 hour in a mixture of goat anti-rabbit 800 (Licor, 926-32211, AB_621843) and goat anti-mouse IgM Alexa Fluor 680 (Jackson ImmunoResearch, 115-625-075, AB_2338934) secondary antibodies each diluted 1:20,000 in non-fat dry milk. The blots were rinsed again and imaged on the Licor system. The signal intensity of the tau bands were quantified and then the signal from the supernatant and pellet fractions were added together to obtain a value representing 100% of the tau signal for each sample set. Then the signals for the supernatant and pellet fractions were expressed as a

percentage of the total tau signal. This experiment was repeated twice with similar results.

Generation and Characterization of MTBR Monoclonal Antibody

We generated a mouse monoclonal antibody against the MTBRs, which was used here to probe the MTBR-GFP proteins on immunoblots. Mice were housed with a normal light cycle at room temperature and humidity with food and water provided *ad libitum*. An adult (10 week-old) female tau knockout mouse (B6.129X1-Mapttm1Hnd/J; Jackson Labs, 007251) was immunized with a tau peptide conjugated to keyhole limpet hemocyanin (KLH-CSTENLKHQPGGGKVQIVYKPVDLSKVT, Genscript) and antibody hybridomas (fusion with SP2/o myeloma cells, Kanaan lab) were generated and screened using methods similar to those described previously by our group[5]. A stable hybridoma line producing an antibody, referred to as the MTBR3 antibody (a mouse IgG1 isotype, AB_2832994), was obtained and subcloned at least 3 times. The MTBR3 antibody displayed strong reactivity against the immunizing peptide (without KLH) and was validated for reactivity against full-length 4R (2N4R) and 3R (2N3R) human tau isoforms using ELISA titer assays as described[5] (Supplementary Fig. 5a). The MTBR3 antibody, was further validated for its utility with immunoblots (the purpose of its use here) using recombinant full-length human 4R and 3R proteins. The ELISA titer and recombinant protein experiments were repeated twice with similar results. All rodent uses/procedures conducted in compliance with federal, state and institutional guidelines and were approved by the Michigan State University Institutional Animal Care and Use Committee (protocol 01/17-004-00).

Supplementary Figures

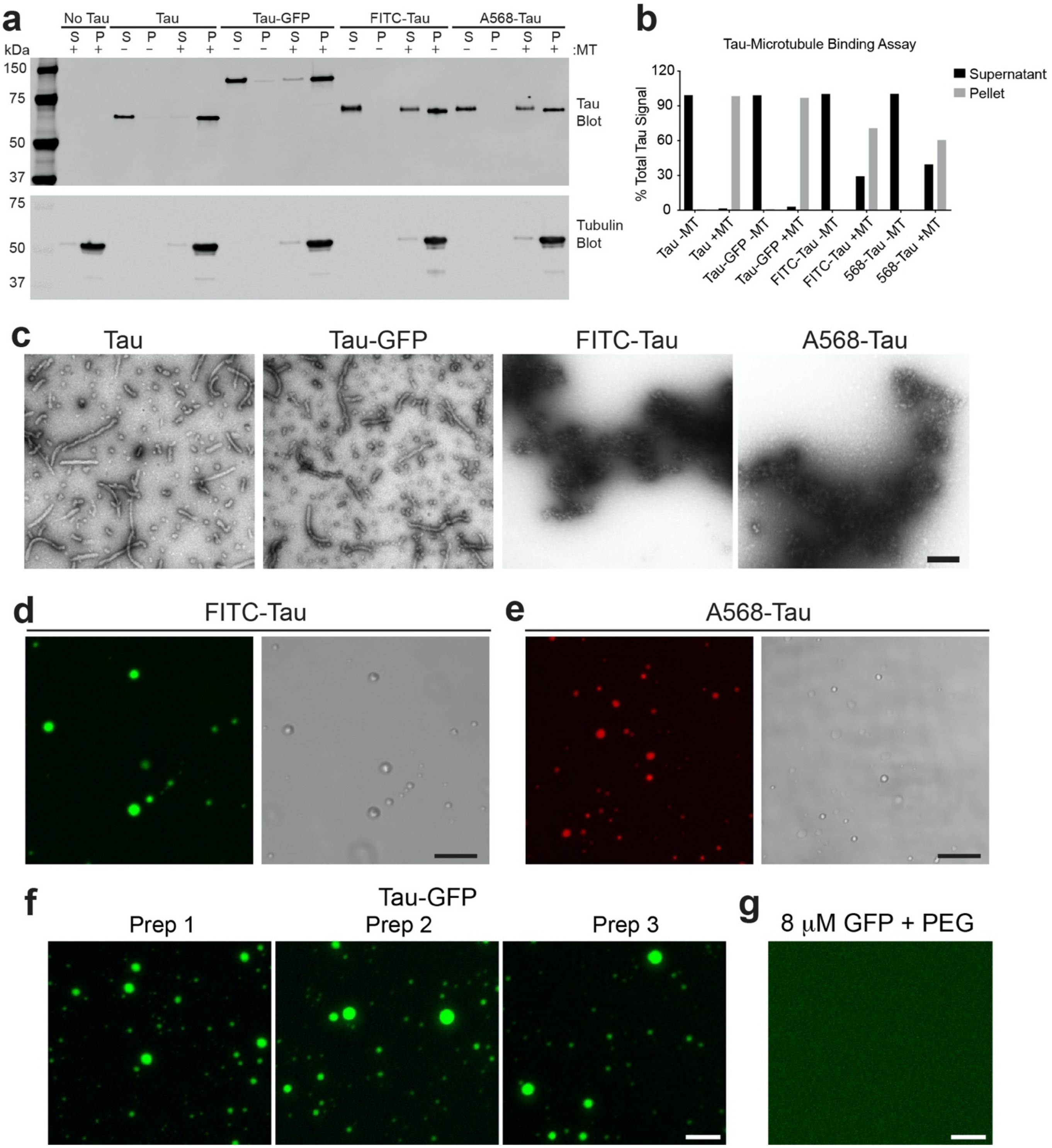

Supplementary Fig. 1. Strategy for studying tau liquid-liquid phase separation. a, Representative blots from a microtubule binding assay to measure binding of Tau, Tau-GFP, FITC-Tau and Alexa568(A568)-Tau proteins. **b,** Quantitation of tau immunoblots

of soluble (S) and pelleted (P) fractions from a representative experiment indicate that Tau and Tau-GFP display the same degree of effective microtubule binding as assessed by their co-pelleting with microtubules (MT), while crosslink labeling of tau with either FITC or A568 impairs microtubule binding. Representative data from one experiment presented, this experiment was repeated two independent times with similar results. **c,** Transmission electron micrographs of Tau and Tau-GFP aggregates (2 μM) formed, which included small globular oligomers and short, intermediate and long filaments typically found with arachidonic acid-induced aggregation *in vitro*. In contrast, FITC-Tau and A568-Tau (2 μM) do not form normal tau aggregate species, but instead form large clumps of protein. Scale bar is 200 nm. These experiments were repeated three independent times. **d, e,** Despite the lack of normal microtubule binding and *in vitro* aggregation properties of FITC-Tau and A568-Tau, both of these labeled proteins undergo liquid droplet formation (4 μM) when incubated with PEG. Scale bar is 10 μm. These experiments were repeated three independent times. **f,** Multiple independent preparations of recombinant Tau-GFP proteins (n = 3) produced the same results confirming that tau phase separation was not due to abnormalities with a single protein preparation. Scale bar is 10 μm. **g,** Incubation of GFP alone (8 μM) with 10% PEG did not produce liquid droplet formation indicating GFP does not undergo phase separation at 2-4 times the amount of tau-GFP constructs used in this study. Scale bar is 10 μm. Source data for panels **a**, **b** provided as a Source Data file.

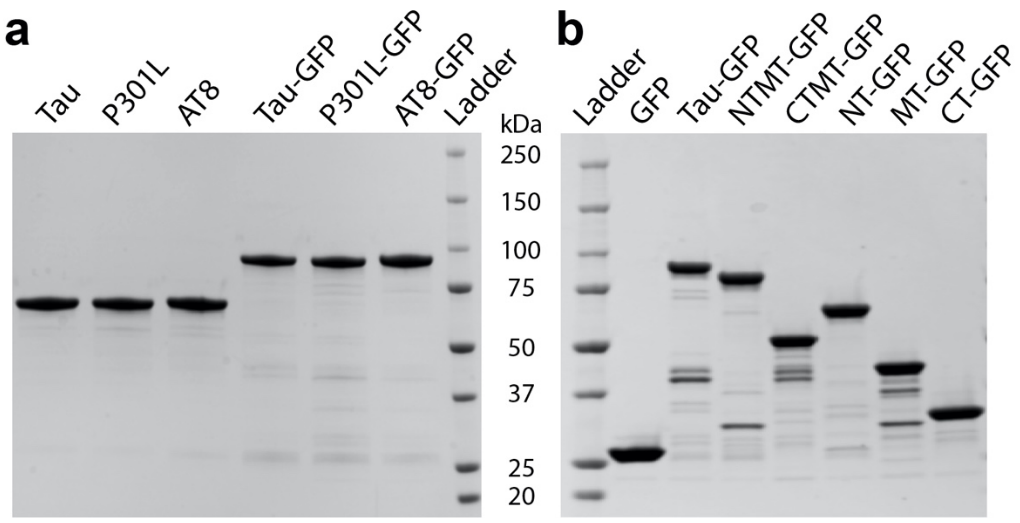

Supplementary Fig. 2 Comparison of each tau construct preparation. a, Unlabeled Tau, P301L, AT8, Tau-GFP, P301L-GFP and AT8-GFP recombinant proteins are comparable to one another as assessed by Coomassie stained SDS-PAGE gel analysis. 3 µg of each protein was loaded per lane. **b**, GFP alone, Tau-GFP, the N-terminus with the microtubule binding repeats (NTMT-GFP; amino acids 1-380), the MTs with the C-terminus (MTCT-GFP; amino acids 225-441), the NT alone (NT-GFP, amino acids 1-224), the MTs alone (MT-GFP; amino acids 225-380), and the CT alone (CT-GFP; amino acids 381-441) recombinant proteins are comparable to one another as assessed by Coomassie stained SDS-PAGE gel analysis. 2 µg of each protein was loaded per lane. Source data for panels **a**, **b** provided as a Source Data file.

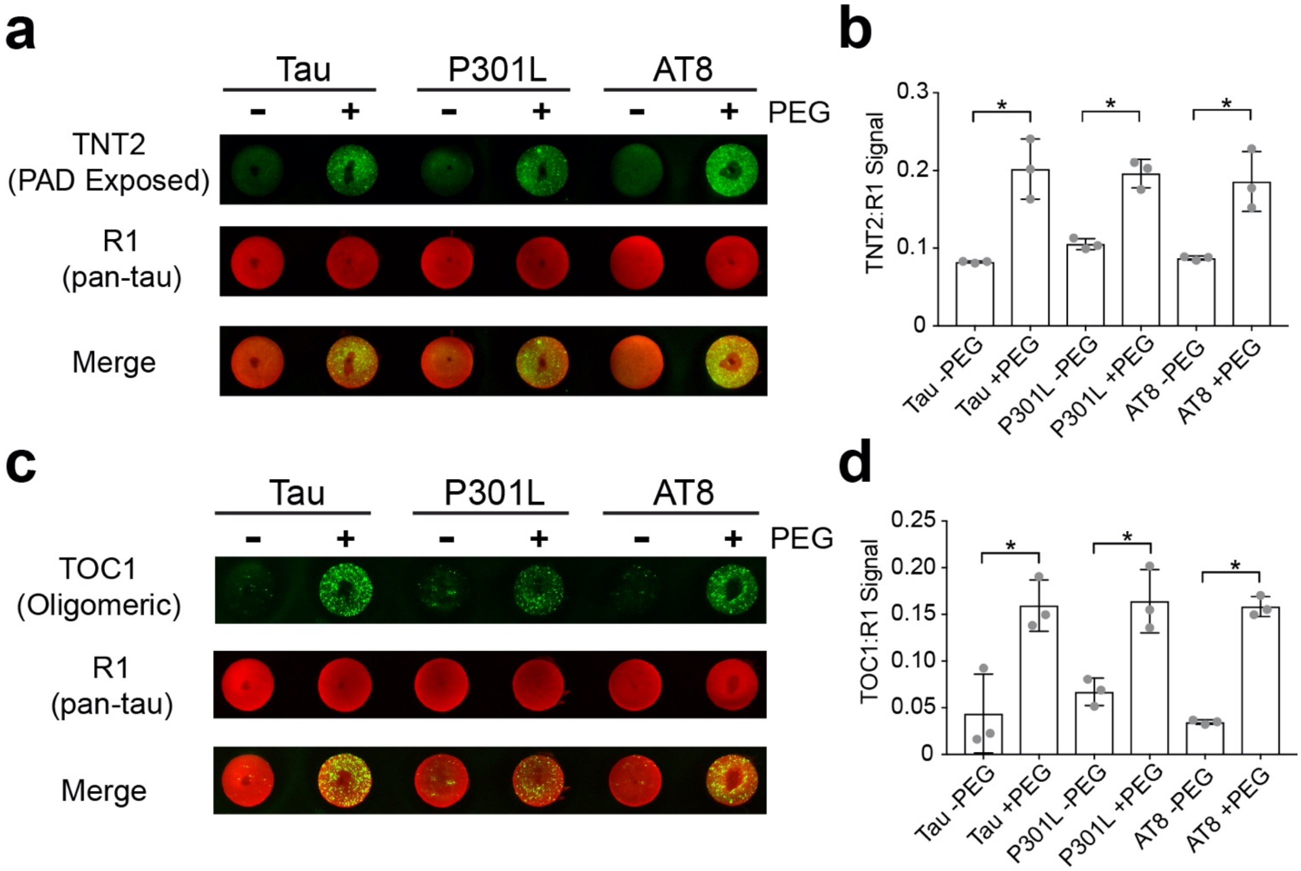

Supplementary Fig. 3. **Formation of pathological tau conformations and oligomers is caused by molecular crowding. a,** Representative dot blots of Tau, P301L and AT8 incubated without (-PEG, monomeric tau) or with PEG (+PEG, liquid droplets) for 4 hours. Blot was probed for total tau (R1 antibody) and PAD-exposed tau (TNT2 antibody). **b,** Quantitation of dot blots show significant increases in PAD exposure upon LLPS of Tau, P301L and AT8 proteins (n = 3 independent experiments, data are mean ±SD, two-way ANOVA with Sidak post-hoc test; Interaction: $F_{(2, 12)}$ = 0.5928, p = 0.5682; Crowding Factor: $F_{(1, 12)}$ = 84.83, p < 0.0001; Tau Factor: $F_{(2, 12)}$ = 0.5449, p = 0.5936). **c,** Representative dot blots of Tau, P301L and AT8 incubated without (–PEG) or with PEG (+PEG) for 4 hours. Blot was probed for total tau (R1 antibody) and oligomeric tau (TOC1 antibody). **d,** Quantitation of dot blots show significant increases in oligomeric tau species upon LLPS of Tau P301L and AT8 (n = 3 independent experiments, data are mean ±SD, two-way ANOVA with Sidak post-hoc test; Interaction: $F_{(2, 12)}$ = 0.4173, p = 0.6681; Crowding Factor: $F_{(1, 12)}$ = 84.26, p <

0.0001; Tau Factor: $F_{(2, 12)} = 0.8712$, $p = 0.4433$). Source data for panels **a-d** provided as a Source Data file.

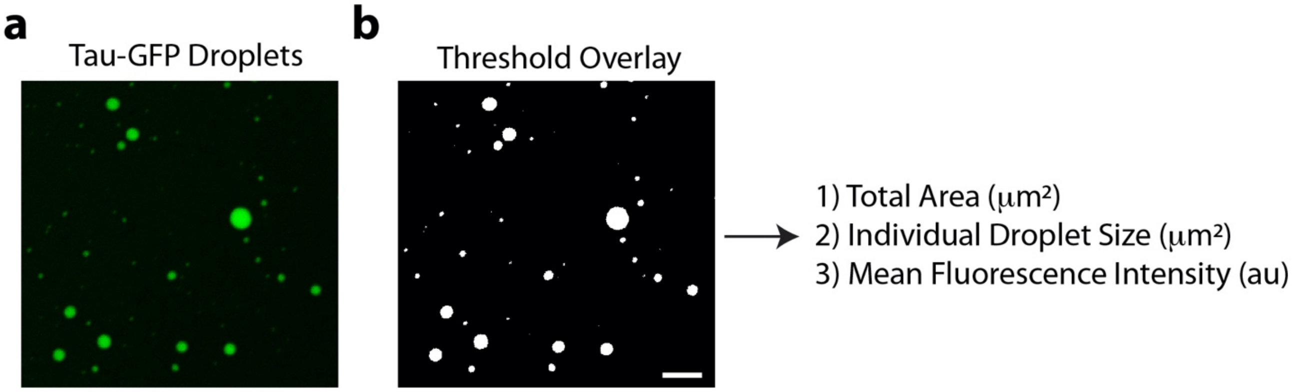

Supplementary Fig. 4. Image analysis of tau liquid droplets. a, Images of tau GFP fusion proteins were acquired on a confocal microscope system. All images within an experiment were acquired using identical confocal settings (scan speed, resolution, magnification, laser intensity, gain, and offset). Scale bar is 10 μm. **b,** Images were analyzed by applying threshold functions in ImageJ software that identified the phase separated tau liquid droplets and excluded the background of the image. All droplets within the threshold limits were analyzed for total area (μm^2), average size of individual droplets (μm^2) and mean fluorescence intensity of individual droplets (arbitrary units).

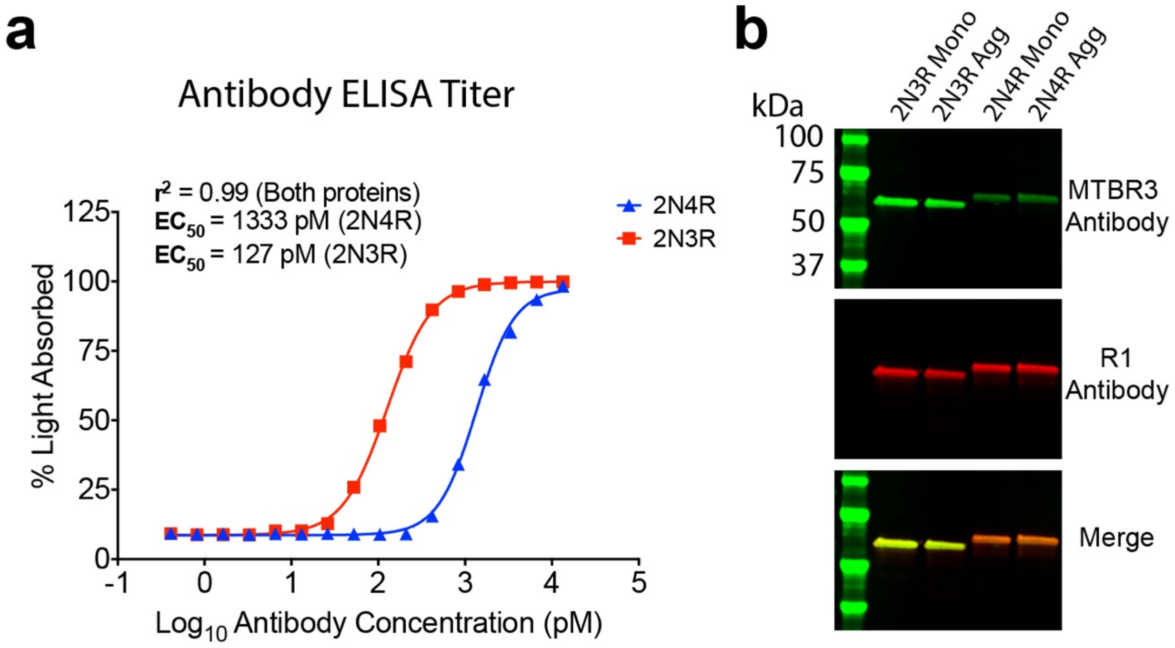

Supplementary Fig. 5. Characterization of MTBR3 antibody. A novel antibody against the MTBR domain of tau was generated by immunizing tau knockout mice with a peptide containing sequences between amino acids 262 and 319 of human tau protein. **a,** The MTBR3 antibody was titered in an ELISA using full-length human 4R tau isoform (2N4R) and 3R tau isoform (2N3R) proteins (representative experiment that was replicated 2 times with similar results, data analyzed using nonlinear regression). **b,** To confirm the MTBR3 antibody reacts with recombinant tau on immunoblots, 2N4R and 2N3R proteins were used (monomeric, Mono and aggregated tau samples, Agg). Tau was confirmed using the polyclonal R1 tau antibody. Note the MTBR3 antibody reacts with both tau isoforms in each assay, but displays a higher affinity for 3R proteins (representative experiment that was replicated 2 times with similar results). Source data for panels **a**, **b** provided as a Source Data file.

Supplementary Table 1. Resources used in this study.

Resource	Source	Catalog #/Reference
Primary and Secondary Antibodies		
Tau12 (mouse IgG1 monoclonal, 1mg/ml stock)	Kanaan Lab	AB_2721192; Ref.[6, 7]
Tau5 (mouse IgG1 monoclonal, 1mg/ml stock)	Kanaan Lab	AB_2721194; Ref.[8, 9]
Tau7 (mouse IgG1 monoclonal, 1mg/ml stock)	Kanaan Lab	AB_2721195; Ref.[10]
MTBR3 (mouse IgG1 monoclonal, 1mg/ml stock)	Kanaan Lab	AB_2832994; This report Supplementary Figure 5.
R1 (rabbit polyclonal, 1mg/ml stock)	L.I. Binder Lab	AB_2832929; Ref.[3]
TNT2 (mouse IgG1 monoclonal, 1mg/ml stock)	Kanaan Lab	AB_2736931; Ref.[6]
TOC1 (mouse IgM monoclonal, 1mg/ml stock)	Kanaan Lab	AB_2832939; Ref.[11, 12]
GFP (rabbit polyclonal, 5mg/ml stock)	Abcam	Cat# ab290; AB_303395
5H1 (mouse IgM monoclonal, 1mg/ml stock)	Kanaan Lab	AB_2832941; Ref.[4]
Goat anti-mouse IgM Alexa Fluor 680 secondary antibody	Jackson ImmunoResearch	Cat# 115-625-075; AB_2338934
Goat anti-mouse IgG1 680 secondary antibody	Licor	Cat# 926-68050; AB_2783642
Goat anti-rabbit 800 secondary antibody	Licor	Cat# 926-32211; AB_621843
ChromPure whole molecule Mouse IgG	Jackson ImmunoResearch	Cat# 015-000-003; AB_2337188
18 nm gold labeled goat anti-mouse IgG (H+L) secondary antibody	Jackson ImmunoResearch	Cat# 115-215-146; AB_2338738
Bacterial Strains		
T7 Express (Recombinant Protein Expression)	New England BioLabs	Cat# C2566I
XL-Gold (DNA Cloning)	Agilent Technologies	Cat# 200315
TOPO® TA Cloning® Kit Dual Promoter (with pCR II-TOPO® vector)	Invitrogen	Cat# K4600-01
Chemicals and Recombinant Proteins		
Recombinant full-length human hT40, 2N4R (Tau)	Kanaan Lab	N/A
Tau with C-terminal GFP (Tau-GFP)	Kanaan Lab	N/A
Recombinant human P301L tau (P301L)	Kanaan Lab	N/A
P301L with C-terminal GFP (P301L-GFP)	Kanaan Lab	N/A
Recombinant human pseudophosphorylated AT8 tau (AT8, S199E/S202E/T205E)	Kanaan Lab	N/A
AT8 with C-terminal GFP (AT8-GFP)	Kanaan Lab	N/A
N-terminal Tau GFP (NT-GFP)	Kanaan Lab	N/A
NT + microtubule binding repeat domain GFP (NTMT-GFP)	Kanaan Lab	N/A
MT alone GFP (MT-GFP)	Kanaan Lab	N/A
MT + C-terminus GFP (MTCT-GFP)	Kanaan Lab	N/A

CT alone GFP (CT-GFP	Kanaan Lab	N/A
GFP alone	Kanaan Lab	N/A
Polyethylene glycol 3000 (expires after 6-8 months)	Sigma	Cat# 81269
Arachidonic acid	Cayman Chemical	Cat# 90010.1
Bovine serum albumin (2 mg/ml)	Thermo Scientific	Cat# 23209
Uranyl acetate	Electron Microscopy Sciences	Cat# 22400
HisLink™ Resin	Promega	Cat# V8823
Thioflavin S	Sigma	Cat# T1892
Commercial Assays		
FluoReporter FITC Labeling Kit	Thermo Scientific	Cat# F6434
Alexa 568 Labeling Kit	Thermo Scientific	Cat# A20184
Microtubule Binding Protein Spin-Down Assay Kit	Cytoskeleton	Cat# BK029
Recombinant DNA Constructs and Supplies		
pT7 Tau constructs (Tau, P301L, AT8, 3R Tau)	Kanaan Lab	N/A
pT7 Tau-GFP and GFP constructs (Tau-GFP, P301L-GFP, AT8-GFP, NT-GFP, NTMT-GFP, MT-GFP, MTCT-GFP, CT-GFP and GFP)	Kanaan Lab	N/A
QuikChange Lightning Site-Directed Mutagenesis Kit	Agilent	Cat# 210519
Platinum™ PCR SuperMix High Fidelity	Thermo Scientific	Cat# 12532024
FastDigest XbaI	Thermo Scientific	Cat# FD0684
FastDigest NdeI	Thermo Scientific	Cat# FD0585
FastDigest EcoRV	Thermo Scientific	Cat# FD0304
FastDigest KpnI	Thermo Scientific	Cat# FD0524
FastDigest NheI	Thermo Scientific	Cat# FD0973
Animals and Eukaryotic Cell Lines		
Female 10 weeks-old mice (B6.129X1-Mapttm1Hnd/J)	Jackson Labs	Cat# 007251
SP2/o myeloma cells	Kanaan Lab	N/A
Computer Software		
NIS Elements Imaging	Nikon	v5.02.00
Licor Imaging Studio	Licor	v5.2.5
SoftMax Pro6	Molecular Devices	v5.2.1
FIJI	Free online download (version 2.0.0-rc-54/1.51f)	http://imagej.net/Fiji/Downloads
GraphPad Prism	GraphPad	v8
Adobe Photoshop	Adobe	v21.1.2
Adobe Illustrator	Adobe	v24.1
JEOL TEM Center	JEOL	v1.5.4.4004
AMT Image Capture Engine	AMT	v602.600.62
Bio-Rad Image Lab	Bio-Rad	v5.2.1
Unicorn Software	GE	v6.4.1.345
AlphaSnap Software	ProteinSimple	v1.4.0.0801
DNAStar SeqMan and SeqBuilder Pro	DNAStar	v15
Microsoft Excel and Word	Microsoft	v16.36
Other Resources		
Nikon A1+ Confocal Microscope System	Nikon	N/A

JEOL 1400+ Transmission Electron Microscope System	JEOL	N/A
Licor Imaging System	Licor	N/A
Whatman dot blot manifold	GE Healthcare	Cat# 10 447 900
8 well coverglass bottom chamber slides	Fisher Scientific	Cat# 12565470
0.22 µm nitrocellulose membrane	VWR	Cat# 27376-991

Supplementary Table 2. Mutagenesis and PCR Primers.

Construct/Strategy	Template DNA	Primers*
**Tau-GFP**: EcoRV-KpnI Insert Mutagenesis (to insert C-terminal GFP with EcoRV-KpnI digestion)	pT7c WT Tau	5'-CTGGCCAAGCAGGGTTTGGATATCGGTACCCATCATCATCATCATCATTGACTCG-3'
**MTCT-GFP**: NdeI Insert Mutagenesis (to remove NT domain with NdeI digestion)	pT7c Tau-GFP	5'-ACCCGGGAGCATATGAAGGTGGCAGTG-3'
**NTMT-GFP**: XbaI-EcoRV Flanked PCR and TOPO cloning (to cut and paste into pT7c Tau-GFP)	pT7c WT Tau	Forward: 5'- TCTAGAAATAATTTTGTTTAACT-3'<br>Reverse: 5'- GATATCCTCGCGGAAGGTCAGCTTGTG-3'
**MT-GFP**: NdeI Insert Mutagenesis (to remove NT domain with NdeI digestion)	pT7c NTMT-GFP	5'-ACCCGGGAGCATATGAAGGTGGCAGTG-3'
**NT-GFP**: EcoRV Insert Mutagenesis (to remove MTCT domain with EcoRV digestion)	pT7c Tau-GFP	5'- CAAGGATATCGGCAGTGGTCCGTACTC-3'
**AT8-GFP**, **P301L-GFP** & **CT-GFP**: Cut and paste cloning	pT7c AT8-His, P301L-His or CT-His (inserts)<br>pT7c Tau-GFP (recipient plasmid)	XbaI (upstream of tau in pT7c) NheI (endogenous at far 3' end of tau) inserts were ligated into pT7c Tau-GFP plasmid

Note: All primers from Integrated DNA Technologies, Inc. and all restriction enzymes from ThermoFisher Scientific.

Supplementary References

1. Combs B, Tiernan CT, Hamel C, Kanaan NM. Production of recombinant tau oligomers in vitro. *Methods Cell Biol* **141**, 45-64 (2017).

2. Cox K, Combs B, Abdelmesih B, Morfini G, Brady ST, Kanaan NM. Analysis of isoform-specific tau aggregates suggests a common toxic mechanism involving similar pathological conformations and axonal transport inhibition. *Neurobiol Aging* **47**, 113-126 (2016).

3. Berry RW*, et al.* Tau epitope display in progressive supranuclear palsy and corticobasal degeneration. *Journal of neurocytology* **33**, 287-295 (2004).

4. Wang Y, Loomis PA, Zinkowski RP, Binder LI. A novel tau transcript in cultured human neuroblastoma cells expressing nuclear tau. *J Cell Biol* **121**, 257-267 (1993).

5. Grabinski T, Kanaan NM. Novel Non-phosphorylated Serine 9/21 GSK3beta/alpha Antibodies: Expanding the Tools for Studying GSK3 Regulation. *Front Mol Neurosci* **9**, 123. eCollection 2016. (2016).

6. Combs B, Hamel C, Kanaan NM. Pathological conformations involving the amino terminus of tau occur early in Alzheimer's disease and are differentially detected by monoclonal antibodies. *Neurobiology of disease*, (2016).

7. Ghoshal N*, et al.* Tau conformational changes correspond to impairments of episodic memory in mild cognitive impairment and Alzheimer's disease. *Experimental neurology* **177**, 475-493 (2002).

8. Carmel G, Mager EM, Binder LI, Kuret J. The structural basis of monoclonal antibody Alz50's selectivity for Alzheimer's disease pathology. *The Journal of biological chemistry* **271**, 32789-32795 (1996).

9. LoPresti P, Szuchet S, Papasozomenos SC, Zinkowski RP, Binder LI. Functional implications for the microtubule-associated protein tau: localization in oligodendrocytes. *Proceedings of the National Academy of Sciences of the United States of America* **92**, 10369-10373 (1995).

10. Horowitz PM, LaPointe N, Guillozet-Bongaarts AL, Berry RW, Binder LI. N-terminal fragments of tau inhibit full-length tau polymerization in vitro. *Biochemistry* **45**, 12859-12866 (2006).

11. Patterson KR*, et al.* Characterization of prefibrillar Tau oligomers in vitro and in Alzheimer disease. *The Journal of biological chemistry* **286**, 23063-23076 (2011).

12. Ward SM, Himmelstein DS, Lancia JK, Fu Y, Patterson KR, Binder LI. TOC1: characterization of a selective oligomeric tau antibody. *Journal of Alzheimer's disease : JAD* **37**, 593-602 (2013).
