# Supplementary material for: Liquid-liquid phase separation induces pathogenic tau conformations in vitro
Source: Nat Commun. 2020 Jun 4;11:2809. doi: 10.1038/s41467-020-16580-3 (PMC7272632; doi:10.1038/s41467-020-16580-3)
Supplement: Supplementary file 2 — Reporting Summary [file 41467_2020_16580_MOESM2_ESM.pdf]

nature research

Corresponding author(s): Nicholas M. Kanaan

Last updated by author(s): Mar 4, 2020

Reporting Summary

Nature Research wishes to improve the reproducibility of the work that we publish. This form provides structure for consistency and transparency in reporting. For further information on Nature Research policies, see Authors & Referees and the Editorial Policy Checklist .

Statistics

For all statistical analyses, confirm that the following items are present in the figure legend, table legend, main text, or Methods section.

n/a	Confirmed	
☐	☒	The exact sample size (*n*) for each experimental group/condition, given as a discrete number and unit of measurement
☐	☒	A statement on whether measurements were taken from distinct samples or whether the same sample was measured repeatedly
☐	☒	The statistical test(s) used AND whether they are one- or two-sided<br>*Only common tests should be described solely by name; describe more complex techniques in the Methods section.*
☒	☐	A description of all covariates tested
☒	☐	A description of any assumptions or corrections, such as tests of normality and adjustment for multiple comparisons
☐	☒	A full description of the statistical parameters including central tendency (e.g. means) or other basic estimates (e.g. regression coefficient) AND variation (e.g. standard deviation) or associated estimates of uncertainty (e.g. confidence intervals)
☐	☒	For null hypothesis testing, the test statistic (e.g. *F*, *t*, *r*) with confidence intervals, effect sizes, degrees of freedom and *P* value noted<br>*Give P values as exact values whenever suitable.*
☒	☐	For Bayesian analysis, information on the choice of priors and Markov chain Monte Carlo settings
☒	☐	For hierarchical and complex designs, identification of the appropriate level for tests and full reporting of outcomes
☒	☐	Estimates of effect sizes (e.g. Cohen's *d*, Pearson's *r*), indicating how they were calculated

Our web collection on statistics for biologists contains articles on many of the points above.

Software and code

Policy information about availability of computer code

Data collection	Nikon Elements Imaging Software (v5.02.00, build 1266, patch 02), Licor Image Studio Software (v5.2.5), SoftMax Pro6 Software (v6.3), JEOL TEM Center Software (v1.5.4.4004), AMT Image Capture Engine Software (v602.600.62), Bio-Rad Image Lab Software (v5.2.1, build 11), GE Unicorn Software (v6.4.1.345), AlphaSnap software (v1.4.0.0801)
Data analysis	Nikon Elements Imaging Software (v5.02.00, build 1266, patch 02), FIJI Image J Software (v2.0), Licor Image Studio Software (v5.2.5), Photoshop (v21.1.2), Illustrator (v24.1), Word and Excel (v16.36), GraphPad Prism (v8.4.1), DNAStar SeqMan (v15), DNAStar SeqBulderPro (v15)

For manuscripts utilizing custom algorithms or software that are central to the research but not yet described in published literature, software must be made available to editors/reviewers. We strongly encourage code deposition in a community repository (e.g. GitHub). See the Nature Research guidelines for submitting code & software for further information.

Data

Policy information about availability of data

All manuscripts must include a data availability statement. This statement should provide the following information, where applicable:

- Accession codes, unique identifiers, or web links for publicly available datasets
- A list of figures that have associated raw data
- A description of any restrictions on data availability

All relevant data will be made available from the corresponding author upon reasonable request.

Field-specific reporting

Please select the one below that is the best fit for your research. If you are not sure, read the appropriate sections before making your selection.

- [x] Life sciences
- [ ] Behavioural & social sciences
- [ ] Ecological, evolutionary & environmental sciences

For a reference copy of the document with all sections, see nature.com/documents/nr-reporting-summary-flat.pdf

Life sciences study design

All studies must disclose on these points even when the disclosure is negative.

Sample size	Sample sizes were not predetermined for these studies. Prior studies have used similar group sizes (n=3-5) with similar biochemical assays (see PMID: 27574109, PMID: 27260838, PMID: 27373205, PMID: 31456657).
Data exclusions	GFP intensity standard curve data were assessed for outlier replicates (>3 times the mean and >2 standard deviations of other replicates), and accordingly a single replicate was removed from the GFP concentration standard curve imaging data (Fig. 2c, see Source Data File). No other replicate or experimental data points were excluded from analysis in this work.
Replication	Experiments were repeated multiple independent times (indicated in the manuscript where relevant). We tested recombinant protein preparation for reproducibility by producing multiple preparations of wild-type tau, all of which displayed similar behaviors (as reported in the manuscript).
Randomization	Randomization is not relevant for this study as the recombinant protein experiments do not involve assignment of samples to different groups. The animal work described here was simply utilization of an animal to generate a new monoclonal antibody, thus,randomization is not possible/necessary.
Blinding	This work involved in vitro recombinant protein assays and the use of an animal to generate a new monoclonal antibody. Blinding was not utilized for the in vitro recombinant protein studies, but controls and group samples were processed either simultaneously or in parallel in all experiments. Binding is not practical or useful in the process of generating a monoclonal antibody and a single animal was used for this purpose.

Reporting for specific materials, systems and methods

We require information from authors about some types of materials, experimental systems and methods used in many studies. Here, indicate whether each material, system or method listed is relevant to your study. If you are not sure if a list item applies to your research, read the appropriate section before selecting a response.

Materials & experimental systems

n/a	Involved in the study	
[ ]	[x]	Antibodies
[ ]	[x]	Eukaryotic cell lines
[x]	[ ]	Palaeontology
[ ]	[x]	Animals and other organisms
[x]	[ ]	Human research participants
[x]	[ ]	Clinical data

Methods

n/a	Involved in the study	
[x]	[ ]	ChIP-seq
[x]	[ ]	Flow cytometry
[x]	[ ]	MRI-based neuroimaging

Antibodies

Antibodies used	Tau12 (mouse IgG1 monoclonal, 1mg/ml stock, Kanaan Lab AB_2721192); Tau5 (mouse IgG1 monoclonal, 1mg/ml stock, Kanaan Lab AB_2721194); Tau7 (mouse IgG1 monoclonal, 1mg/ml stock, Kanaan Lab AB_2721195); MTBR3 (mouse IgG1 monoclonal, 1mg/ml stock, Kanaan Lab AB_2832994); R1 (rabbit polyclonal, 1mg/ml stock, L.I. Binder Lab, AB_2832929); TNT2 (mouse IgG1 monoclonal, 1mg/ml stock, Kanaan Lab AB_2736931); TOC1 (mouse IgM monoclonal, 1mg/ml stock, Kanaan Lab, AB_2832939); GFP (rabbit polyclonal, 5mg/ml stock, Abcam, Cat# ab290; AB_303395); 5H1 (mouse IgM monoclonal, 1mg/ml stock, Kanaan Lab AB_2832941); Goat anti-mouse IgM Alexa Fluor 680 secondary antibody (Jackson ImmunoResearch Cat# 115-625-075, AB_2338934); Goat anti-mouse IgG1 680 secondary antibody (Licor Cat# 926-68050; AB_2783642); Goat anti-rabbit 800 secondary antibody (Licor Cat# 926-32211; AB_621843); ChromPure whole molecule Mouse IgG (Jackson ImmunoResearch Cat# 015-000-003; AB_2337188); 18 nm gold labeled goat anti-mouse IgG (H+L) secondary antibody (Jackson ImmunoResearch Cat# 115-215-146; AB_2338738).
Validation	The majority of antibodies are well-established antibodies and/or commercially available antibodies. Tau12 (PMID: 27260838, PMID: 12429193); Tau5 (PMID: 7479786; PMID: 8955115); Tau7 (PMID: 17042504); MTBR3 (this report, Supplementary Figure 5); R1 (PMID: 15475684); TNT2 (PMID: 27260838); TOC1 (PMID: 21550980; PMID: 23979027); 5H1 (PMID: 8468346); GFP (https://www.abcam.com/gfp-antibody-chip-grade-ab290.html); Goat anti-mouse IgM Alexa Fluor 680 secondary antibody

(https://www.jacksonimmuno.com/catalog/products/115-625-075); Goat anti-mouse IgG1 680 secondary antibody (https://www.licor.com/bio/reagents/irdye-680lt-goat-anti-mouse-igg1-specific-secondary-antibody); Goat anti-rabbit 800 secondary antibody (https://www.licor.com/bio/reagents/irdye-800cw-goat-anti-rabbit-igg-secondary-antibody); ChromPure whole molecule Mouse IgG (https://www.jacksonimmuno.com/catalog/products/015-000-003); 18 nm gold labeled goat anti-mouse IgG (H+L) secondary antibody (https://www.jacksonimmuno.com/catalog/products/115-215-146).

Eukaryotic cell lines

Policy information about cell lines

Cell line source(s)	SP2/o myeloma cell (Kanaan lab).
Authentication	This line was not authenticated beyond successful utilization in forming viable hybridoma cell lines.
Mycoplasma contamination	All hybridoma lines were tested for mycoplasma and are free of such contamination.
Commonly misidentified lines (See ICLAC register)	No commonly misidentified lines were used here.

Animals and other organisms

Policy information about studies involving animals; ARRIVE guidelines recommended for reporting animal research

Laboratory animals	An adult (10 week-old) female tau knockout mouse (B6.129X1-Mapttm1Hnd/J, Jax) was used to generate novel anti-MTBR tau antibody (housing conditions described in supplementary methods section).
Wild animals	This study did not involve wild animals.
Field-collected samples	This study did not include field-collected samples.
Ethics oversight	The Michigan State University Institutional Animal Care and Use Committee approved this animal work.

Note that full information on the approval of the study protocol must also be provided in the manuscript.
